# Androgen receptor splice variants and prostate cancer: From bench to bedside

**DOI:** 10.18632/oncotarget.14537

**Published:** 2017-01-06

**Authors:** Kristine M. Wadosky, Shahriar Koochekpour

**Affiliations:** ^1^ Department of Cancer Genetics, Center for Genetics and Pharmacology, Roswell Park Cancer Institute, Buffalo, NY, USA; ^2^ Department of Urology, Center for Genetics and Pharmacology, Roswell Park Cancer Institute, Buffalo, NY, USA

**Keywords:** prostate cancer, AR splice variant, castration-resistant, clinical, molecular biology

## Abstract

Therapeutic interventions for advanced prostate cancer (PCa) center on inhibiting androgen receptor (AR) and downstream signaling pathways. Resistance to androgen deprivation therapy and/or AR antagonists is inevitable and molecular mechanisms driving castration-resistant PCa (CR-PCa) primarily involve alterations in AR expression and activity. Detailed molecular biology work over the past decade, discussed at length in this review article, has revealed several AR transcripts that result from alternative splicing. These AR splice variants are increased in cell and mouse models of CR-PCa and in CR-PCa tumors. Several AR variants lack the ligand binding domain, but retain their ability to bind DNA and activate transcriptionlinking constitutive AR function and therapeutic failure. ARV7 is the only variant endogenously detected at the protein level and thus has undergone more thorough molecular characterization. Clinical trials in PCa are currently investigating ARV7 utility as a biomarker and new therapeutics that inhibit ARV7 . Overall, this review will illustrate the historical perspectives of AR splice variant discovery using fundamental molecular biology techniques and how it changed the clinical approach to both therapeutic decisions and strategy. The body of work investigating AR splice variants in PCa represents a true example of translational research from bench to bedside.

Prostate cancer (PCa) is the leading diagnosed non-cutaneous cancer among men in the United States, as estimated by the American Cancer Society in 2016 [1]. Identification and clinical staging of prostatic neoplasms is performed with a combination of diagnostic tests, including digital rectal examination, serum prostatic specific antigen (PSA), and histological analysis of tumor biopsies with grading according to the Gleason system [2–9]. Most newly diagnosed PCa cases are low-risk and require minimal therapeutic intervention that is primarily curative [10]. Some low-risk PCa patients even elect for active surveillance, which entails giving curative treatment only after evidence of disease progression, such as increasing PSA levels [11]. For approximately 8% of patients, PCa is diagnosed as advanced or metastatic—where additional intervention is needed to control disease progression [1,12, 13]. For 75 years, the primary non-surgical treatment strategy for advanced PCa has been androgen deprivation therapy (ADT) [13–15]. These treatments have remained at the forefront of disease management for PCa because androgen hormones, with the primary androgen being testosterone, are potent stimulators of PCa growth and survival [13, 14, 16]. Unfortunately, ADT is not curative and most patients will relapse within 2 years despite castrate levels of serum testosterone (50 ng/dL); where disease recurrence is defined as raising PSA levels on 2 subsequent occasions with possible evidence of tumor progression *via* imaging analysis (bone scan, computated tomography (CT), or magnetic resonance imaging (MRI)) or repeat biopsies [2, 3, 17–19]. These patients are then considered to have castration-resistant PCa (CR-PCa), a highly advanced form of PCa that is lethal within about 2 years [20]. It was originally thought that CR-PCa is independent of androgens; however, FDA approval of CR-PCa drugs within the last 5 years that target the androgen axis has eradicated this notion [13, 21–24]. Therefore, treatment options for CR-PCa patients include abiraterone, which inhibits a pivotal enzyme in steroid hormone synthesis, or enzalutamide, which is an antagonist of androgen receptor (AR) [21–24]. As discussed in detail below, AR is a transcription factor that activates genes which promote PCa cell proliferation and survival [16]. In recent years, resistance to these second-generation ADT agents has also emerged—leaving these CR-PCa patients to be treated with chemotherapeutic agents docetaxel or cabazitaxel [25–29]. Each of the four CR-PCa treatment options increase patient survival by merely months [25]. Therefore, the urgency to understand the mechanisms by which PCa become resistant to both first- and second-generation ADT has been at the forefront of both basic and clinical research in PCa. Considering that ADT limits production of androgen hormones and AR activity, a large subset of studies have focused on AR-dependent mechanisms of PCa progression [25].

## AR IN PROSTATE CANCER

**Figure 1:**
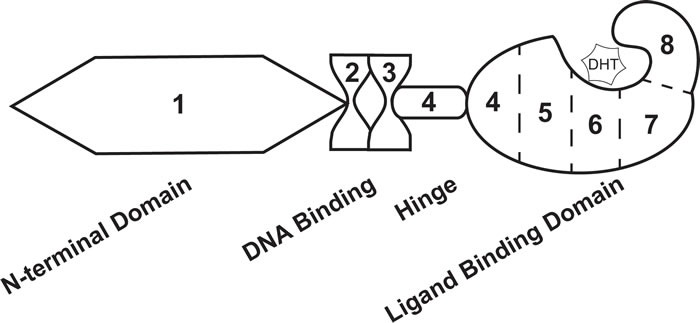
Androgen receptor exon and protein structure The androgen receptor transcript is comprised of 8 exons that codes for 4 distinct protein domains. Exon 1 codes for the N-terminal domain, exons 2 and 3 code for the DNA binding domain, exon 4 codes for the hinge domain, and exons 4-8 code for the ligand binding domain. Dihydrotestosterone (DHT) interacts with the ligand binding domain of androgen receptor.

In addition to being exploited in treatment schemes for advanced PCa, androgens take part in molecular signaling pathways that impact PCa disease progression. AR is a nuclear receptor transcription factor that is directly activated by androgen hormones [13, 16]. The gene that codes for AR is located on the X chromosome at location Xq11-12 [13, 30]. Eight exons code for the AR protein that is 919 amino acids in length and 110 kDa [13, 30]. The protein structure of AR is equivalent to that of other nuclear receptors, including the N-terminal domain (NTD) encoded by exon 1, DNA-binding domain (DBD) encoded by exons 2 and 3, hinge domain (HD) encoded by exon 4, and ligand-binding domain (LBD) encoded by exons 4-8 [13, 30] (Figure 1). Notably, the LBD binds dihydrotestosterone (DHT), a potent AR ligand synthesized when testosterone enters the cell [13] (Figure 1). Upon stimulation, AR translocates to the nucleus where it binds to androgen response elements (AREs) in the promoter regions of androgen response genes (ARGs) [16]. Regulation of AR activity can occur at the protein level, including interaction with regulator proteins and post-translational modifications , and the genomic level, including several key mutations linked to PCa [25, 30–32]. Within the past decade, several groups have demonstrated that AR activity is also regulated by alternative splicing—where AR splice variants (ARVs) that lack the LBD promote constitutive gene transcription in the absence of androgen hormones [33]. Expression of ARVs have been a foremost mechanism used to explain persistent AR activity, PCa cell survival, and disease progression with ADT [25]. In addition, the discovery of ARVs has had a large clinical impact, where this basic finding is now being utilized in biomarker development to predict CR-PCa patient response to therapy [34–36]. In this review article, we take a thorough approach to describing the key basic molecular biology responsible for the identification of ARVs, the pre-clinical data that support ARV's involvement in human PCa, and the clinical studies that led to the proposition for use of ARVs as prognostic biomarkers. We propose that ARVs are a prime example of translational uro-oncology research that has truly progressed from “bench to bedside.”

## EARLY INDICATIONS OF THE EXISTENCE OF AR SPLICE VARIANTS

First evidence of endogenous expression of ARVs in PCa cell lines was published in the early 2000s by one of the groups that originally cloned AR. In this work, lower molecular weight bands between 80-90 kDa were detected by AR immunoblot and these bands were more pronounced in CR-PCa cell lines [37]. It was further deduced using antibodies against different protein regions of AR that these variants lacked the LBD [37]. At the time, it was hypothesized that these variants derived from proteolytic cleavage of AR , but a large body of work produced since has shown that they result from alternative mRNA splicing [37–39].

**Figure 2:**
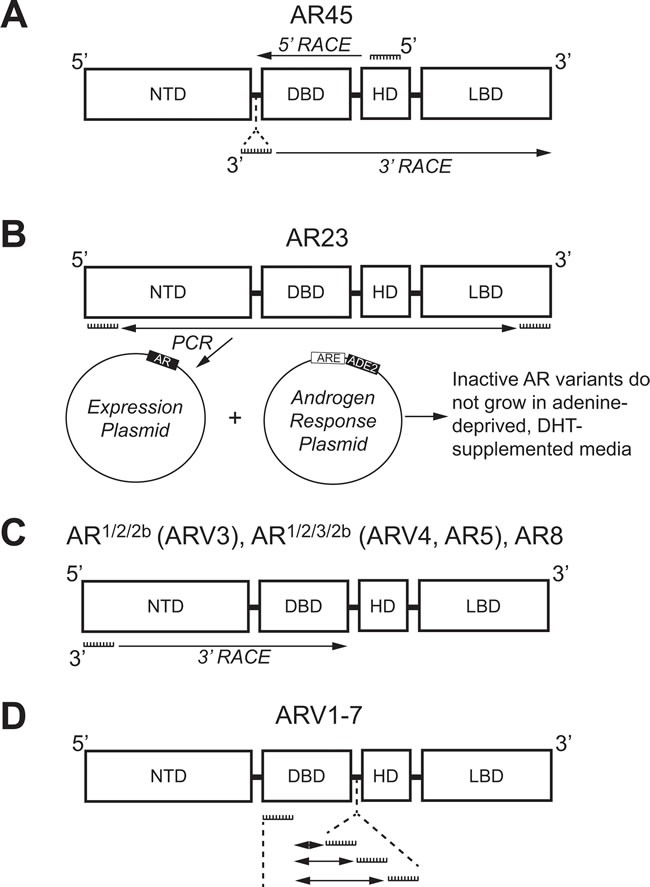
Methologies used to identify androgen receptor splice variants AR45, AR23, AR 1/2/2b, AR1/2/3/2b, AR8, and ARV1-7. **A**. 5’-RACE with a primer against the coding region for the HD (Exon 4) and 3’-RACE with a primer against a unique N-terminal intronic region were used to identify AR45. **B**. Primers against the coding region for the NTD (Exon 1) and LBD (Exon 8) were used for PCR and subcloning into a yeast expression plasmid. Co-transformation with plasmid containing an adenine production gene (ADE2) under control of an ARE identified the clone expressing inactive AR23 using adenine-depleted medium supplemented with DHT. **C**. 3’-RACE using primers against the coding region for the NTD (Exon 1) was employed to identify AR^1/2/2b^, AR^1/2/3/2b^, and AR8. **D**. Multiple primer sets, each containing a primer against the coding region for the DBD (Exon 2) and a primer against one of three different intronic cryptic exons (CE1-3), were used to identify ARV1-7 using PCR. AR: Androgen receptor; ARE: Androgen response element; ARV: Androgen receptor splice variant; CE: Cryptic exon; DBD: DNA binding domain; DHT: Dihydrotestosterone; NTD: N-terminal domain; HD: Hinge domain; LBD: Ligand binding domain; RACE: Rapid amplification of DNA ends.

**Figure 3:**
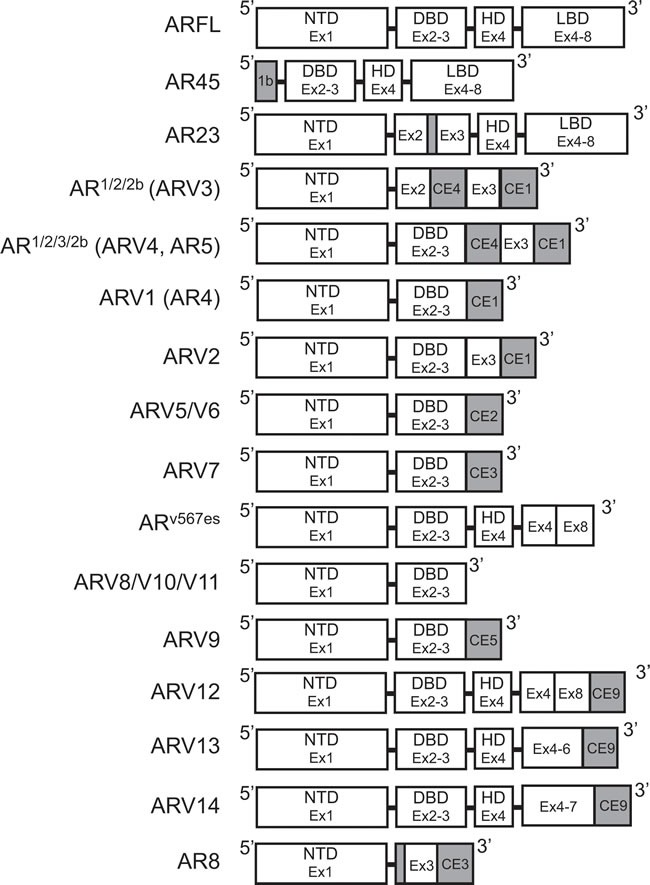
Structure of androgen receptor splice variants Exon and protein domain structure for known androgen receptor splice variants. White: Sequence corresponds to AR-FL structure. Gray: Unique sequences. AR: Androgen receptor; AR-FL: Androgen receptor full length; ARV: Androgen receptor splice variant; CE: Cryptic exon.

Despite the multitude of ARVs lacking the LBD, the very first studies that investigated individual ARVs discovered those with the LBD intact. In 2005, an alternative AR mRNA transcript was identified with 5’ rapid amplification of cDNA ends (5’-RACE) [40]. With a forward primer anchored in the AR HD (Figure 2A), 5’-RACE generated a product much shorter in length than expected [40]. Sequencing determined that this product contained the coding regions for the DBD, but only a short unique set of nucleotides where the coding region for the NTD was expected to be (Figure 3) [40]. RACE in the opposite direction (3’-RACE) using a forward primer against this unique N-terminal sequence (Figure 2A), called exon 1b, determined that this newly-discovered AR transcript contained the full coding sequences for the DBD, HD, and LBD (Figure 3) [40]. This alternative AR transcript, named AR45 for its protein product size of 45 kDa, was expressed in normal prostate tissue and LNCaP cells (Table 1); however, it was most highly expressed in skeletal and cardiac muscle [40]. Activation of AR45 by the synthetic androgen R1881 was minimal in a mouse mammary tumor virus (MMTV)-driven luciferase assay when expressed in PC3 cells, an AR-negative PCa cell line [40]. In addition, it was shown that AR45 could possibly act as a dominant negative by interacting with wildtype AR (Table 1) [40].

**Table 1:** Androgen Receptor Splice Variants containing the Ligand Binding Domain

Variant	Endogenous Expression	Exons	Protein Regions	Activity Status	References
AR45	LNCaP	1b, 2, 3, 4, 5, 6, 7, 8	Partial NTD, DBD, Hinge, LBD	Inactive, dominant negative	[34]
AR23	Not determined	1, 2, 3, 4, 5, 6, 7, 8	NTD, interrupted DBD, Hinge, LBD	Inactive, cytoplasmic	[35]

AR: Androgen receptor; NTD: N-terminal domain; DBD: DNA binding domain; LBD: Ligand-binding domain

Using a yeast-based functional assay, another ARV transcript was identified in 2007 from a CR-PCa bone metastasis [41]. In this assay, a yeast androgen-inducible expression system was established using a plasmid containing an ARE upstream of ADE2 (Figure 2B), a gene which allows yeast to grow in the absence of adenine [42]. Therefore, when a second plasmid containing wildtype full-length AR is co-transformed with the androgen response plasmid, yeast colonies containing both plasmids will grow in medium lacking adenine and supplemented with DHT (Figure 2B) [42]. Using cDNA from tumor tissue isolated from the bone marrow aspirate of a CR-PCa patient, the AR coding region was cloned with a forward primer against exon 1 and reverse primer against exon 8 (Figure 2B) [41, 42]. The AR expression plasmid prep, containing any AR cDNA sequences amplified using those primers, was co-transformed with the androgen response plasmid and cultures were grown and plated with fully supplemented medium [42]. Individual colonies were then tested by culturing them in medium lacking adenine and supplemented with DHT—those colonies that grew were considered to express a plasmid containing the wildtype, full-length AR (AR-FL) sequence, but those that did not presumably expressed plasmid coding for a form of AR that is not activated by DHT [42]. The plasmids from colonies that did not grow were isolated and sequenced, the results of which led to the discovery of an ARV transcript with a 69 nucleotide insertion between exon 2 and 3 (Figure 3) [41]. This unique sequence codes for a set of 23 amino acids inserted in the DBD (Figure 3) that interferes with the two zinc finger structures that enable AR to bind to DNA (Figure 1) [13, 41]. Known as AR23, overexpression of this variant in LNCaP cells showed that it is unable to activate transcription *via* an ARE-driven luciferase assay [41]. In addition, AR23 was exclusively cytoplasmic and unable to translocate to the nucleus (Table 1) [41]. Despite this interesting work, AR23 has not been shown to be endogenously expressed in any PCa cell lines (Table 1) [38, 39]. A drawback to this study is that only AR transcripts containing the wildtype exon 1 and exon 8 sequences recognized by the primers could be cloned (Figure 2B) —therefore, any transcripts lacking exon 1 or 8, many of which have been discovered (Tables 2 and 3), were missed using this assay [42]. Altogether, these early studies of ARVs lacked the impact of later studies since both AR45 and AR23 are inactive variants and unlikely to contribute to ADT resistance (Table 1). Nevertheless, identification of these variants set the foundation for future studies that have proven to be highly relevant to understanding the molecular pathogenesis of CR-PCa.

**Table 2:** Androgen Receptor Splice Variants lacking the Ligand Binding Domain

Variant	Endogenous Expression	Exons	Protein Regions	Activity Status	References
ARV7 (AR3)	22Rv1, LNCaP (mRNA only), C-81, C4-2, C4-2B, LNCaP 95, VCaP, CWR-R1	1, 2, 3, CE3	NTD, DBD	Ligand-independent, Nuclear	[38, 39, 42, 43, 47]
AR^v567es^	VCaP	1, 2, 3, 4, 8	NTD, DBD, Hinge	Ligand-independent, Nuclear	[41, 46, 47]
ARV12	22Rv1	1, 2, 3, 4, 8, CE9	NTD, DBD, Hinge	Ligand-independent, Nuclear	[43]
AR^1/2/2b^ (ARV3)	22Rv1, LNCaP, VCaP, LAPC4	1, 2, CE4, 3, CE1	NTD, Partial DBD	Ligand-independent	[37, 38]
AR^1/2/3/2b^ (ARV4, AR5)	22Rv1, CWR-R1	1, 2, 3, CE4, 3, CE1	NTD, DBD	Ligand-independent	[37–39]
ARV1 (AR4)	22Rv1, CWR-R1, VCaP	1, 2, 3, CE1	NTD, DBD	LNCaP: Ligand-independent, PC3: Inactive, Cytoplasmic	[38, 39, 43, 47]
ARV9	22Rv1, VCaP	1, 2, 3, CE5	NTD, DBD	LNCaP: Ligand-independent, PC3: Inactive, Cytoplasmic	[43, 47]
ARV2	22Rv1	1, 2, 3, 3, CE1	NTD, DBD	Not determined	[38]
ARV5/V6	22Rv1	1, 2, 3, CE2	NTD, DBD	Not determined	[38]
ARV8/10/11	VCaP	1, 2, 3	NTD, DBD	Not determined	[47]

AR: Androgen receptor; NTD: N-terminal domain; DBD: DNA binding domain; LBD: Ligand-binding domain

**Table 3:** Other Androgen Receptor Splice Variants

Variant	Endogenous Expression	Exons	Protein Regions	Activity Status	References
AR8	22Rv1, CWR-R1, C4-2, C4-2B	1, 3, CE3	NTD, partial DBD	Inactive, Membrane	[50]
ARV13	22Rv1	1, 2, 3, 4, 5, 6, 9	NTD, DBD, Hinge, Partial LBD	Inactive	[43]
ARV14	22Rv1	1, 2, 3, 4, 5, 6, 7, 9	NTD, DBD, Hinge, Partial LBD	Not determined	[43]

AR: Androgen receptor; NTD: N-terminal domain; DBD: DNA binding domain; LBD: Ligand-binding domain

## METHODOLOGIES LEADING TO AR SPLICE VARIANT DISCOVERY

### AR splice variants without the ligand binding domain

It was not until the work of Dr. Scott Dehm from the laboratory of Dr. Donald Tindall in 2008 that the study of ARVs in PCa began in earnest [43]. With a forward primer against AR exon 1 (Figure 2C), 3’ RACE led to the discovery of two distinct transcripts that contained exon 1, 2, and a novel nucleotide sequence downstream of either exon 2 or exon 3, known as exon 2b or cryptic exon 4 (CE4) [43]. These variants, named AR^1/2/2b^ and AR^1/2/3/2b^, were discovered in 22Rv1 cells that have tandem duplication of exon 3 (Table 2) ; therefore, it was determined that CE4 is located in the intronic region between exon 2 and 3 (Figure 3) [30, 43]. Indeed, AR^1/2/3/2b^ has only been detected in either 22Rv1 or CWR-R1 cells (Table 2), derived from the same CWR22 parent cell line, both by Dehm *et al*. and other groups [43–45]. The NTD and at least part of the DBD are coded for by the AR^1/2/2b^ and AR^1/2/3/2b^ transcripts (Table 2), therefore these ARV proteins can bind DNA in the absence of androgens [43]. Indeed, only siRNA against the AR NTD was capable of completely inhibiting basal AR activity in 22Rv1 cells, as measured by a MMTV-luciferase assay [43]. Evidence of the constitutive activity of AR^1/2/2b^ or AR^1/2/3/2b^ was shown when their expression in the AR-negative PCa cell line DU145 equally induced MMTV-luciferase activity independent of treatment with the potent AR ligand, mibolerone [43]. Finally, AR^1/2/2b^ was detected by real-time PCR (RT-PCR) in LNCaP, VCaP, and LAPC4 PCa cell lines, demonstrating that expression of this constitutively-active ARV is a universal phenomenon [43].

Both Hu *et al*. and Guo *et al*. , published within a year after Dehm *et al*. , verified the presence of ARVs in PCa cells [43–45]. Using Basic Local Alignment Search Tool (BLAST^®^), Hu *et al*. searched for *AR* intronic sequences in the human expressed sequence database [44]. This methodical *in silico* genomic search method identified transcribed sequences that PCR and subsequent sequencing established as CE1-4 (Figure 3) [44]. As stated above, CE4 was previously identified as exon 2b [43], but CE1-3 between AR exon 3 and 4 were novel [44]. With a forward primer against exon 2 and reverse primers against CE1, CE2, or CE3 (Figure 2D), seven distinct ARVs were amplified using cDNA generated from 22Rv1 cells [44]. These ARVs were named ARV1-7 and all lacked the LBD because of premature stop codons upstream of exon 4 (Table 2, Figure 3) [44]. ARV3 and ARV4 in Hu *et al*. were nearly identical to AR^1/2/2b^ and AR^1/2/3/2b^ , respectively, except each also contained CE1 at their 3’ end (Table 2, Figure 3) [43, 44]. Therefore ARV1, ARV2, ARV5, ARV6, and ARV7 were novel splice variants identified by Hu *et al*.; however ARV5 and ARV6 only slightly differ in their 3’ sequences downstream of exon 3 (Table 2) [44].

Only ARV1 and ARV7 were further characterized by Hu *et al*. since their data for these variants suggested that they were more widely expressed and likely to display constitutive activity than ARV2, AR^1/2/3/2b^/ARV4, AR^1/2/2b^/ARV3, and ARV5/V6 [44]. Quantitative RT-PCR for ARV1 and ARV7 in 9 different PCa cells lines determined that ARV1 is expressed in VCaP and 22Rv1 and ARV7 is expressed in LNCaP, LNCaP 95, VCap, and 22Rv1 (Table 2) [44]. Neither ARV1 nor ARV7 were expressed in LAPC4, MDA-PCa2b, E006AA, PC3, and DU145 [44]. These data suggested that ARV7 was the predominant ARV. The authors went on to use the unique peptide sequence coded for by CE3 to generate an ARV7-specific antibody (Figure 3) [44]. ARV7 immunoblot showed protein bands at the predicted molecular weight of ARV7 (75kDa) in VCaP and 22Rv1 cells, but not in LNCaP or PC3 [44]. Interestingly, despite LNCaP expressing ARV7 at the transcript level, ARV7 protein could not be detected [44]. Nevertheless, these data were the first to show that an ARV transcript is translated in PCa cells [44]. Separation of cytoplasmic and nuclear extracts in VCaP and 22Rv1 cells showed that ARV7 is localized to the nucleus even in the absence of androgen—suggesting that endogenous ARV7 is an active transcription factor and constitutively-active (Table 2) [44]. Altogether, the findings of Hu *et al*. confirmed the original discovery of AR^1/2/2b^/ARV3 and AR^1/2/3/2b^/ARV4, identified novel ARVs, and most significantly, established that ARV7 is endogenously expressed at the protein level [44]. Since its original discovery, the importance of ARV7 has been strengthened—ARV7 has been the most consistently expressed variant in recurrent PCa cell lines, CR-PCa tissue samples, and pre-clinical PCa models of castration resistance [38, 39].

The discovery of AR^1/2/3/2b^/ARV4, ARV1, and ARV7 in PCa cells was also confirmed by Guo *et al*. [45]. Evidence of three different ARV transcripts in CWR-R1 cells was shown using 3’ RACE with an AR exon 1 primer (Figure 2C) [45]. This study named these variants AR3, AR4, and AR5, but they have the same sequences as ARV7, ARV1, and AR^1/2/3/2b^/ARV4, respectively (Table 2, Figure 3) [43, 44]. Importantly, this study was the first to show that ARV7, ARV1, and AR^1/2/3/2b^/ARV4 are expressed in CWR-R1 cells (Table 2) [45]. It was also shown that ARV7 was the most highly expressed variant by RT-PCR in LNCaP, C-81 (a high passage recurrent derivative of LNCaP), CWR-R1, and 22Rv1 cells, again suggesting that ARV7 is the primary ARV [45]. Guo *et al*. also produced an antibody against ARV7, different only by the use of a longer peptide sequence containing an additional 7 amino acids upstream of the peptide used for the antibody in Hu *et al*. [44, 45]. The 75kDa ARV7 protein was detected by immunoblot in C-81, C4-2, C4-2B, CWR-R1, and 22Rv1, where expression of ARV7 was highest in 22Rv1 cells [45]. Again, ARV7 protein was not expressed in LNCaP cells; however, C-81, C4-2, and C4-2B, LNCaP-derived cell lines that are insensitive to androgen deprivation, did express enough ARV7 protein to be detected by immunoblot [45]. In addition, a recent report by our group verified that ARV7 is expressed in 22Rv1 and CWR-R1 cells, but not in their isogenic primary androgen-sensitive cell line CWR22Pc [46]. These data provide further evidence of ARV7 expression in resistance to androgen deprivation. Together, these data imply that enhanced expression of ARV7 protein by these cells could be a way by which they maintain growth in the absence of androgens [44, 45].

### Exon-skipping AR splice variants

**Figure 4:**
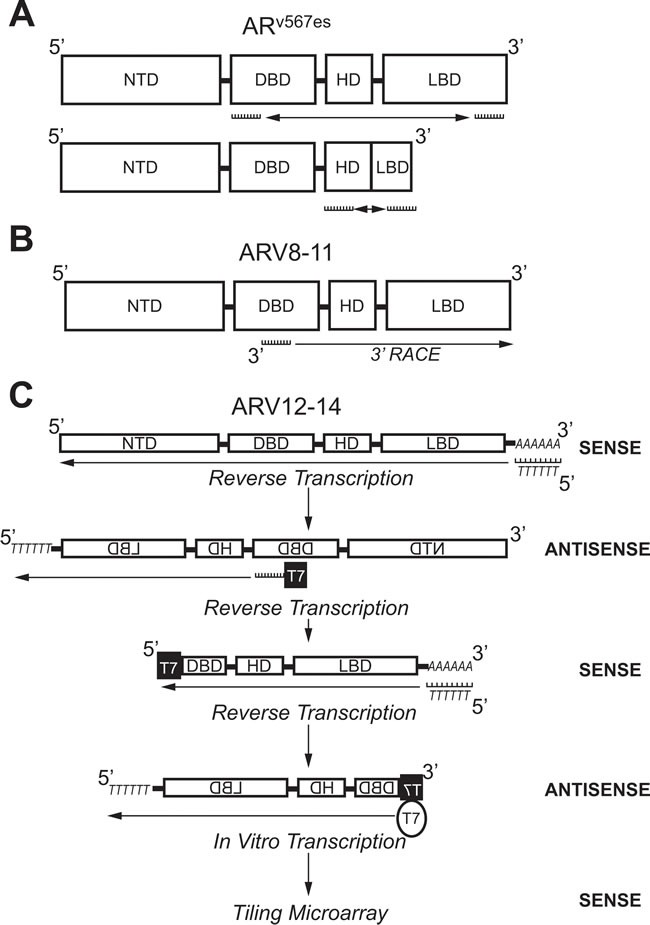
Molecular biology methods used to identify androgen receptor splice variants AR v567es, ARV8-11, and ARV12-14. **A**. Primers against the coding region for the DBD (Exon 2) and LBD (Exon 8) were used to amplify AR^v567es^ using PCR. Confirmation of exon-skipping was done using PCR with primers against coding regions for the HD (Exon 4) and LBD (Exon 8). **B**. A primer against the coding region for the DBD (Exon 2/3) was used in 3’-RACE to identify ARV8-11. **C**. Selective linear amplification of sense RNA (SLASR) was used to identify ARV12-14. After reverse transcription using an oligo-dT primer, reverse transcription was repeated with a primer against the coding region for the DBD (Exon 3) containing the T7 RNA polymerase consensus sequence. A third round of reverse transcription was then performed using an oligo-dT primer. Finally, T7 RNA polymerase was used to amplify DBD-containing transcripts and a tiling microarray performed to identify ARV12-14. AR: Androgen receptor; ARV: Androgen receptor splice variant; DBD: DNA-binding domain; NTD: N-terminal domain; HD: Hinge domain; LBD: Ligand binding domain; RACE: Rapid amplification of DNA ends; SLASR: Selective linear amplification of sense RNA.

ARV transcripts have also been found which originate from exon skipping during mRNA splicing. RT-PCR using primers against AR exon 2 and 8 (Figure 4A) showed shorter PCR products than predicted in 2 out of 25 cDNAs from LuCaP xenografts [47]. Sequencing of these PCR products showed that they both coded for identical AR transcripts that lack exons 5, 6, and 7 ; consequently, this ARV was named AR^v567es^ (Table 2) [47]. When primers were designed to amplify the junction between exon 4 and 8 (Figure 4A), products were detected in almost all of the original LuCaP xenografts, showing that AR^v567es^ is universally expressed in the LuCaP model [47]. Despite containing the sequence for exon 8, a frame shift that occurs as a result of exon skipping leads to a premature stop codon [47]. Therefore, AR^v567es^ contains codons for only 10 amino acids coded for by exon 8 (Figure 3) [47]. By lacking the LBD, AR^v567es^ is constitutively-active—as determined with an ARE luciferase assay performed in the AR-negative M12 PCa cell line [47]. Several groups have shown that AR^v567es^ can be translated when it is exogenously expressed , but there are no AR^v567es^-specific antibodies available that recognize endogenous protein levels [38, 39, 47–51]. Peacock *et al*. suggest that bands below 75 kDa observed in VCaP xenografts *via* AR immunoblot with an NTD-specific antibody represents endogenous protein expression of AR^v567es^ , but without a variant-specific antibody, this claim cannot be substantiated [52]. Nevertheless, considering that several studies have confirmed AR^v567es^ expression in PCa clinical samples , AR^v567es^ is considered second-most to ARV7 in relevance to ARV-dependent mechanisms of resistance [38, 39, 47, 49, 53, 54].

Next-generation sequencing (NGS) has also been used to identify ARVs. After 3’ RACE using VCaP cDNA with a primer anchored at the border on AR exons 2 and 3 (Figure 4B), products were submitted to either traditional cloning/Sanger sequencing or NGS [53]. These methods identified 6 ARVs in total, where 2 were previously discovered (ARV1 and ARV7) and 4 were novel (ARV 8-11) (Table 2) [53]. In addition, the NGS data alluded to the presence of ARV transcripts produced as a result of exon skipping ; confirming the discovery of AR^v567es^ by Sun *et al*. [47, 53]. An additional CE (named CE5) within intron 3 between CE2 and CE3, was identified downstream of exons 1-3 in the ARV9 transcript (Table 2, Figure 3) [53]. ARV8, ARV10, and ARV11 also contained exons 1-3, but each had a unique downstream 3’ sequence coded for by intron 3 (Table 2) [53]. As with the majority of other ARVs, ARV9 and ARV8/10/11 all lack the LBD (Table 2) [53]. A strength of NGS is that the relative abundance of sequencing reads can be assessed—as expected, the number of reads for *AR* exons was far greater than those that corresponded to the CEs and unique sequences in ARV transcripts [53]. These data confirmed that expression of ARVs is far lower than AR-FL, as others have shown by conventional methods [43–45].

While 5’ and 3’ RACE do not restrict the amplification of transcripts to two specific primer sequences, the method utilizes a single primer and is still considered biased [55]. To identify ARVs *via* an unbiased method, Hu *et al*. designed a protocol using *in vitro* transcription by T7 RNA polymerase [49]. In this method, named selective linear amplification of sense RNA (SLASR), three rounds of cDNA synthesis was performed with RNA isolated from 22Rv1 cells or CR-PCa tissue samples (Figure 4C) [49]. The first round of cDNA synthesis was performed using an oligo-dT primer to generate antisense sequences of all transcripts containing a poly-A tail (Figure 4C) [49]. For the second round of cDNA synthesis, a fusion primer was used containing the T7 RNA polymerase promoter sequence and sequence corresponding to AR exon 3 to specifically generate sense cDNA for AR transcripts containing exon 3 and sequences downstream of exon 3 [49]. The third round of cDNA synthesis was performed using an oligo-dT primer again to amplify exon 3-containing antisense AR transcripts now containing the T7 consensus sequence (Figure 4C) [49]. Finally, 3^rd^ strand cDNAs were submitted to *in vitro* transcription using T7 RNA polymerase to generate sense cDNA products with the AR exon 3 sequence located at the terminal 5’ end (Figure 4C) [49].

The AR cDNA products resulting from SLASR were submitted to a tiling microarray with 60-nucleotide probe sequences corresponding to the full *AR* locus [49]. This method confirmed the presence of AR transcripts containing CE 1-4, as well as those transcripts derived from exon skipping (Table 2 , Figure 3) [49]. In addition, AR transcripts amplified *via* the SLASR method also contained 3’ regions that corresponded to sequences downstream of AR exon 8—this novel exon was named exon 9 (Figure 3) [49]. ARV12-14 discovered *via* the SLASR method contained exon 9 and lack one or more LBD exons (Table 2, Figure 3) [49]. Further analysis showed that other than having exon 9 located at its 3’ end, the sequence for ARV12 was the same as the sequence for AR^v567es^ (Table 2) [49]. Other similarities between ARV12 and AR^v567es^ include their constitutive activity and nuclear localization (Table 2) [49]. ARV13 lacked exon 7 and 8 and ARV14 lacked only exon 8, while both also contained exon 9; consequently, ARV13 and ARV14 were inactive (Table 3, Figure 3) [49]. Since ARV12 (AR^v567es^) is constitutively active, it is most likely to contribute to ADT resistance, while it is improbable that ARV13 and ARV14 are involved in development of CR-PCa [49]. This study demonstrates that ARVs with partial LBDs are not equivalent; where lacking exons 7 and 8 (ARV13) or exon 8 (ARV14) renders the variant inert, while lacking exons 5, 6, and 7 leads to constitutive activity (ARV12/AR^v567es^) [47, 49].

### Other AR splice variants

Perhaps the most unique of the AR variants discovered to date is AR8, which has been shown to be exclusively associated with the plasma membrane (Table 3) [56]. Using 3’-RACE with a primer anchored in AR exon 1 and cDNA generated from CWR-R1 cells (Figure 2C), a short AR transcript was discovered that coded for only exon 1, 3, and CE3 (Table 3, Figure 3) [56]. Interestingly, upstream of exon 3, AR8 contains the same 69 nucleotide sequence identified in the non-active AR23 variant (Figure 3) [39, 56]. RT-PCR using an AR8-specific primer set showed that AR8 is also expressed at the message level in LNCaP, C4-2, and C4-2B cell lines [56]. Co-immunofluorescence experiments using antibodies against endogenous AR and exogenous FLAG-tagged AR8 in LNCaP cells showed that AR8 is associated with the plasma membrane and does not interact with endogenous AR [56]. Further analysis of AR8 protein structure showed that two cysteine residues in the unique C-terminal region are capable of being palmitoylated and that mutation of these amino acids resulted in loss of AR8 membrane association [56]. It is still unclear what role AR8 plays in the development of therapy resistance in PCa, since it is neither activated by androgens nor functions as an active transcription factor [56]. However, the authors have suggested that AR8 promotes signaling downstream of epidermal growth factor (EGF) by interacting with EGFR and thereby activates canonical activity of AR-FL [56].

## BIOLOGICAL ACTIVITIES OF AR SPLICE VARIANTS IN VITRO

### Transcriptional profiles promoted by AR splice variants

Many of the studies described above used luciferase assays with exogenous promoters to characterize ARV activity. To determine the ability of ARV7 to activate endogenous transcription, Hu *et al*. overexpressed ARV7 in LNCaP cells and performed a gene expression microarray for ARGs [44]. ARV7-overexpressing LNCaP cells activated 20 different ARGs, including transmembrane protease, serine 2 (*TMPRSS2*), FK506 Binding Protein 5 (*FKBP5*), kallikrein-related peptidase 2 (*KLK2*), and *KLK3*, to a similar extent as R1881-treated LNCaP cells [44]. These data show that ARV7's constitutive activity in luciferase assays also applies to endogenous transcription [44]. Another study confirmed these data using quantitative RT-PCR to show that overexpression of ARV7 in LNCaP cells activates transcription of *TMPRSS2* and *FKBP51* in the absence of androgen stimulation [48]. Transcriptional activation of *TMPRSS2* and *FKBP51* was also observed in LNCaP cells overexpressing AR^v567es^ [47]. In addition, AR^v567es^ activated expression of several genes not classified as ARGs—suggesting that distinct genes are regulated by ARVs [47]. With these data in mind, another study sought to characterize gene transcription profiles for ARVs [57]. In this study, either AR-FL or ARV7 was overexpressed in LNCaP cells and RNA was submitted to comprehensive gene expression microarray analysis [57]. Changes in expression of cell cycle genes specifically occurred in cells overexpressing ARV7, while genes whose expression changed in AR-FL overexpressing cells included those involved in biosynthesis, metabolism, and secretion [57]. These gene enrichment sets were defined as ARV7 UP and AR-FL UP, respectively [57]. As proof of principle, the authors showed that the expression pattern of the well-characterized ARGs, *KLK3*, *TMPRSS2*, *NKX3.1*, followed that of AR-FL UP [57]. When either ARV7 or AR^v567es^ was overexpressed in LNCaP cells with stable knockdown of AR-FL, ARV7 UP was enriched—indicating that ARV transcriptional activity could be independent of AR-FL [57]. These data were the first to show the downstream functional independence of ARVs from AR-FL, since other reports suggested that ARVs required AR-FL to activate transcription [47, 53].

### DNA-binding activities of AR splice variants

Transcriptional studies *via* microarray or RT-PCR provide insight into the overall effect of a transcription factor on gene expression. Differences in expression of a specific gene could result from a transcription factor binding its promoter or could result from indirect effects of this transcription factor on other transcription factors and/or DNA-binding proteins. Tangible proof of a transcription factor's direct effect on a specific gene requires data showing that the transcription factor binds to the region of DNA corresponding to the gene's loci. One method that determines a protein's endogenous DNA-binding activity is chromatin immunoprecipitation (ChIP), which immunoprecipitates a DNA-binding protein of interest from nuclear lysates, isolates DNA bound to that protein, and analyzes bound DNA by PCR or sequencing. Using ChIP and subsequent semi-quantitative PCR, Guo *et al*. showed in both 22Rv1 and CWR-R1 cells that ARV7 binds ARE sequences within the promoter region of *AKT1*, a well-characterized oncogene [45]. In addition to being the first direct evidence of ARV7 binding to DNA, this study compared ARV7 ChIP with AR-FL ChIP and found that unlike ARV7, AR-FL did not bind the *AKT1* promoter [45]. Conversely, when the promoter region of *PSA* was analyzed, AR-FL ChIP was positive for this region, as expected, while ARV7 ChIP was not [45]. Together, these data suggest that while ARVs bind promoter regions of canonical ARGs, they can also bind promoter regions of other unique genes.

To ascertain if ARV7 is dependent on AR-FL for its DNA binding activity, Cao *et al*. conducted a study to determine if both AR-FL and ARV7 bind promoter regions of either *PSA* or *UBE2C* [51]. *PSA* served as a canonical ARG and *UBE2C* served as an ARV7-specific gene since its expression is increased with ARV7 overexpression in microarray analysis [51, 57]. ARV7 ChIP was performed in 22Rv1 cells, which was followed by re-ChIP for AR-FL and quantitative PCR for the promoter regions of *PSA* or *UBE2C* [51]. ChIP-PCR showed that both ARV7 and AR-FL occupy the *PSA* promoter under basal conditions, in the presence of DHT, and following treatment with enzalutamide [51]. These data show that interaction of ARV7 with AR-FL at the promoter region of a canonical ARG induces constitutive transcriptional activity, even in the presence of an AR inhibitor [51]. PCR performed for the *UBE2C* promoter using DNA isolated from ARV7 ChIP/AR-FL re-ChIP did not amplify this DNA region, showing that both ARV7 and AR-FL do not occupy this promoter [51]. When ARV7 ChIP was performed in 22Rv1 cells with AR-FL knockdown, the PCR results were positive for the *UBE2C* promoter—showing that ARV7 occupies this promoter alone and does not require AR-FL to do so [51]. Finally, when ARV7 ChIP with AR-FL knockdown or AR-FL ChIP with ARV7 knockdown was performed, amplification of the *PSA* promoter using the immunoprecipitated DNA was decreased compared to non-targeting controls for both experiments [51]. These data show that ARV7 and AR-FL are mutually dependent on each other when occupying the *PSA* promoter [51]. Of note, the same *PSA* promoter primers utilized by Guo *et al*., which were unable to show that ARV7 bound to the *PSA* promoter by semi-quantitative PCR , were able to detect the *PSA* promoter region in the DNA from ARV7 ChIP by quantitative PCR [45, 51]. Together, these ChIP studies by Cao *et al*. show that ARV7 heterodimerizes with AR-FL at the *PSA* promoter, suggesting that canonical ARGs are constitutively regulated by both AR-FL and ARV7 in cell lines that express ARV7 [51]. In addition, ARV7 ChIP also showed that ARV7 binds the *UBE2C* promoter without AR-FL, demonstrating that ARV7 can regulate gene transcription of non-ARGs independently of AR-FL [51].

The development of NGS technology has provided molecular biologists with a way to analyze ChIP-bound DNA without the need for specific primers. This method is referred to as ChIP sequencing (ChIP-seq) and is a powerful tool since it compiles an unbiased set of DNA regions occupied by a specific DNA-binding protein. A CWR-R1-derived cell line (R1-AD1) was recently used in the laboratory of Dr. Scott Dehm to engineer a novel cell line that lacks AR exons 5, 6, and 7 (R1-D567) [58]. Since R1-D567 cells exclusively express AR^v567es^ , these cells can be used to perform molecular biology experiments on AR^v567es^ in the absence of an AR^v567es^-specific antibody [58]. AR^v567es^ ChIP-seq was performed using nuclear lysates from R1-D567 with an AR NTD-specific antibody and ChIP-seq for AR-FL was performed using the same antibody in the parental R1-AD1 cells treated with DHT [59]. Results from AR-FL ChIP-seq in DHT-treated R1-AD1 cells showed 12030 AR-FL DNA binding sites, while AR^v567es^ ChIP-seq in R1-D567 cells showed only 3554 AR^v567es^ DNA binding sites [59]. The number of binding sites containing ARE sequences were similar for both AR-FL and AR^v567es^ ChIP-seq data [59]. There were 1031 common binding sites for AR-FL and AR^v567es^, which included those that were bound with the highest affinity by either transcription factor [59]. There were 2523 unique AR^v567es^ binding sites identified with ChIP-seq in R1-D567 cells; however, the majority of these sites were determined to be false-positives because they were located in regions of repetitive DNA sequences [59]. In addition, the presence of AR^v567es^-specific binding sites could not be repeated in independent ChIP-seq experiments; therefore, it was concluded that AR^v567es^ does not bind unique loci [59]. Considering the multiple studies described above which identified specific genes activated and bound by ARVs , the results of the AR^v567es^ ChIP-seq in R1-D567 cells were unexpected [45, 51, 57, 59]. Consequently, the authors consolidated ChIP-seq reads for the three replicates to augment signal intensity and manually searched for genes shown by others to be “ARV-specific” [57, 59]. Sequencing reads were not found within 100kb of any of these ARV-specific genes in the AR^v567es^ ChIP-seq data [59]. However, AR-FL and AR^v567es^ ChIP-seq datasets did show that AR-FL and AR^v567es^ co-occupy several genes within the ARV-specific list, including *UBE2C* [59]. The authors concluded from this data that AR^v567es^ does not bind to unique gene loci and instead binds canonical ARE sequences in an AR-FL dependent manner [59]. While these data are seemingly in opposition to the ChIP studies performed by Guo *et al*. and Cao *et al*. , these studies showed that unique genes were bound by ARV7 *via* ChIP; therefore it is possible that the nature of ARV7 and AR^v567es^ transcriptional activity and dimerization are different [45, 51]. As described below, the molecular details of homodimerization and heterdimerization of AR^v567es^ are indeed distinct from that of ARV7 [60].

A recent study determined the molecular nature of homodimerization and heterodimerization of ARVs and AR-FL using bimolecular fluorescence complementation (BiFC) in live cells [60]. In this method, a fusion protein is constructed for each possible interaction partner so that one protein within the pair contains the N-terminal of the Venus fluorescent protein and the other protein within the pair contains the C-terminal of the Venus fluorescent protein [60]. A fluorescence signal is detected when the two fusion proteins interact because the complete Venus fluorescent protein is established [60]. Fusion protein plasmid constructs were made for AR-FL, ARV7, and AR^v567es^ with the Venus fluorescent protein N- and C-terminal fragments [60]. Since there are specific regions within both the AR NTD and DBD (Figure 1) that govern AR-FL homodimerization , the authors also generated BiFC fusion protein plasmids for AR-FL, ARV7, and AR^v567es^ that harbor mutations in each or both of these domains [60, 61]. The AR-negative PC3 PCa cell line was transfected with pairs of the BiFC fusion protein plasmid constructs for AR-FL, ARV7, or AR^v567es^ and nuclear fluorescence was determined using fluorescent microscopy [60]. A BiFC signal was detected in nuclei of cells transfected with AR-FL and AR^v567es^ fusion plasmids, showing that AR^v567es^ heterodimerizes with AR-FL in the nucleus ; these data confirm the results of the ChIP-seq that show that AR^v567es^ and AR-FL co-occupy certain gene loci [59, 60]. In addition, data from this study also confirmed the reports from the ARV7 ChIP/AR-FL re-ChIP studies ; where PC3 cells transfected with ARV7 and AR-FL fusion plasmids showed positive BiFC nuclei, indicating that ARV7 and AR-FL heterodimerize in the nucleus [51, 60]. It was shown that both the NTD and DBD dimerization motifs were required for heterodimerization of AR^v567es^ /AR-FL and ARV7/AR-FL, since mutation of both these motifs was needed to inhibit BiFC signals in cells transfected with AR^v567es^ or ARV7 and AR-FL fusion constructs [60]. BiFC studies also showed that ARV7 homodimerizes in the nucleus of PC3 cells , as expected based on the ARV7 ChIP data [51, 60]. In addition, AR^v567es^ homodimerizes both in the nucleus and the cytoplasm based on BiFC signal [60]. AR^v567es^ and ARV7 also heterodimerize and this BiFC signal was also detected both in the cytoplasm and the nucleus [60]. For ARV7 homodimers, AR^v567es^ homodimers, and AR^v567es^ /ARV7 heterodimers, mutation of the DBD dimerization motif, but not the NTD dimerization motif, inhibited BiFC signal from these interaction partners [60]. These data suggest that ARVs dimerize *via* their DBD domains only, unlike ARVs and AR-FL which heterodimerize *via* both their DBD and NTD domains [60].

It is important to note, that while detection of BiFC signal in nuclei suggest that the homodimerization and heterodimerization interactions described above occur while AR-FL, ARV7, and AR^v567es^ are bound to endogenous DNA, these data make this assumption based on cellular localization only [60]. Indeed, it was observed that AR^v567es^ forms homodimers and heterodimers with ARV7 in the cytoplasm and the nucleus, suggesting that these interactions are independent of DNA [60]. The authors performed luciferase assays to show that dimerization is required for transcriptional activity of ARV7 and AR^v567es^ at exogenous promoters , but did not perform ChIP/re-ChIP or ChIP-seq to define AR-FL, ARV7, and AR^v567es^ homodimerization and heterodimerization in terms of specific endogenous genes [60]. Nevertheless, this study confirmed known and identified novel dimerization interactions between ARVs and ARVs/AR-FL using a cell imaging method yet to be utilized in the study AR molecular biology [60]. In addition, dimerization of ARVs was dependent on different protein domains (DBD only) than dimerization of ARVs/AR-FL (NTD and DBD) (Figure 1), providing evidence to show that ARVs not only activate transcription, but also interact with other ARVs in a different way than with AR-FL [60].

## AR SPLICE VARIANTS IN PRE-CLINICAL MODELS OF PROSTATE CANCER

The benefit of *in vitro* cell culture lies with the relative ease an experimental system can be manipulated by drugs, recombinant viruses, DNA/RNA transfection, culture techniques, and live imaging. While the simplicity of cell culture enables researchers to pinpoint specific pathways or proteins responsible for an effect described at the cellular level, it also raises uncertainties about the potential clinical utility of these data. *In vivo* studies of tumor biology, most often carried out in the mouse, more accurately approximate human disease than *in vitro* cell culture because of biological factors that exist independently from cancer cells which can affect cell growth and survival [62, 63]. Some of these factors include interaction with non-cancer cells, such as fibroblasts and immune cells, presence of extracellular matrix, development of microvasculature *via* angiogenesis, and effects of soluble factors, such as hormones and cytokines [62, 63]. *In vivo* study of PCa at the pre-clinical level can be carried out using human xenografts, both cell line-derived xenografts and patient-derived xenografts (PDX), and genetically-engineered mouse models (GEMMs) [64]. Using these models, several research groups have shown that ARVs are expressed *in vivo* and play a role in PCa tumor growth, progression, and therapy resistance.

### AR splice variants in cell line-derived human xenograft models of prostate cancer

To establish a cell line-derived xenograft, cells are first grown in 2D cell culture. Once cell density has reached optimal levels to produce concentrated suspensions, these cells are combined with matrigel and injected subcutaneously into an immunocompromised mouse. Tumor volume can then be routinely monitored using simple caliper measurements [62]. Guo *et al*. achieved forced expression of ARV7 in the LNCaP cell line, which does not basally express ARV7, by transducing cells with an ARV7-expressing lentivirus [45]. Xenografts established with ARV7-expressing LNCaP cells were significantly larger than those established with LNCaP cells transduced with control lentivirus starting at 4 weeks post-grafting and continuing through weeks 5 and 6 [45]. Conversely, two cell lines that basally express ARV7, 22Rv1 and CWR-R1, were transduced with a lentivirus expressing shRNA against ARV7 to stably knockdown ARV7 [45]. As expected, tumor volume of xenografts with ARV7 knockdown were significantly decreased at 3, 4, and 5 weeks post-grafting compared to control xenografts for both 22Rv1 and CWR-R1 [45]. Together, these data show that ARV7 expression directly correlates with PCa tumor growth for human xenografts established with either an androgen-dependent PCa cell line (LNCaP) or CR-PCa cell lines (22Rv1 and CWR-R1) [45]. Similarly, an independent study showed that xenografts established with LNCaP cells transduced with an ARV7-expressing lentivirus were significantly larger than control xenografts when grafted in pre-castrated mice [53]. These data suggest that the constitutive activity of ARV7 is retained *in vivo* by promoting tumor growth in an androgen-depleted environment [53]. Unlike ARV7-expressing LNCaP xenografts, ARV1-expressing LNCaP xenografts were not significantly larger than control xenografts in pre-castrated mice, indicating that ARV1 does not promote tumor growth *in vivo* [53]. Using a different *in vivo* xenografting method, one study established orthotopic xenografts by combining VCaP cells with medium and injecting the mixture into the dorsolateral prostate of immunocompromised mice [65]. Unfortunately, the authors did not accurately measure tumor size using imaging methods, such as with MRI , but monitored tumor progression using serum PSA measurements only [65,66]. VCaP orthotopic prostate xenografts were allowed to grow until serum PSA levels reached ≥ 10 ng/mL, after which mice were castrated and PSA monitored [65]. PSA levels decreased following castration, as expected; when PSA regained levels similar to those prior to castration, tumors were considered to have recurred and were harvested [65]. Expression of ARV1 and ARV7 was measured in tumor tissue by quantitative RT-PCR, showing that expression of both variants was significantly increased in CR tumors compared to intact tumors [65]. While this study did not apply methods standard to the field of *in vivo* PCa research, these data show that mRNA expression of ARVs in human xenografts established in the prostatic microenvironment are increased following development of ADT resistance [65].

Several other studies have shown that expression of either ARV7 or AR^v567es^ correlate with castration recurrence. Subcutaneous VCaP xenografts were established in intact mice and allowed to grow to 500-1000 mm^3^, at which time mice were castrated and tissue was collected at 2, 5, 14, and 22 days post-castration [53]. For VCaP xenografts from intact mice, protein expression of ARVs was not detected by immunoblot , but ARV bands between 60 and 80 kDa with an AR NTD antibody were apparent in VCaP xenografts from castrated mice even after only 2 days [53]. ARV protein expression in VCaP xenografts increased in a time-dependent manner at 5, 14, and 22 days post-castration [53]. When mice were castrated for 14 days, then supplemented with testosterone for the following 8 days using a subcutaneous pellet, VCaP xenografts had decreased ARV protein expression [53]. In fact, ARV protein levels in testosterone-supplemented VCaP xenografts were to levels similar to intact mice [53]. These data further support the negative correlation between ARV expression and testosterone levels [53].

### AR splice variants in human cell line-derived xenografts treated with second generation ADT

*In vivo* xenograft studies using second generation ADT also show that ARV expression is increased in tumors resistant to these new agents. Yu *et al*. established VCaP xenografts and when tumors reached approximately 1 cm^3^, mice were castrated and tumors monitored until volume was restored to ~ 1 cm [67, 68]. Mice with CR tumors were then treated with abiraterone (0.5 mg/mL) in their drinking water for 4-6 weeks until tumors relapsed again [67]. Biopsies were obtained from intact, castrated (4 days post-castration), and CR xenografts [67]. Quantitative RT-PCR analysis showed that ARV7 expression increased with each stage of tumor progression [67]. Importantly, quantitative RT-PCR using RNA isolated from VCaP xenografts pre- and post-abiraterone treatment showed that expression of ARV7 was also increased in abiraterone-resistant tumors [67]. Expression of AR-FL was increased as well, but fold increases at the primary tumor to CR and CR to abiraterone-resistant stages were minimal compared to fold increases in ARV7 expression [67]. These data show that ARV7 expression not only correlates with development of resistance to initial androgen deprivation, but also with resistance to second generation ADT, suggesting a causative role for ARV7 in continued failure of PCa therapies [67].

Similarly, expression of ARVs is also correlated with response to the AR antagonist enzalutamide and development of enzalutamide resistance in cell-derived xenograft models. 22Rv1 cells were transduced with ARV7 shRNA-expressing lentivirus and xenografts were established in intact mice [51]. Once tumors reached 100 mm^3^, mice were treated with enzalutamide by oral gavage (10 mg/kg/day) [51]. Compared to control 22Rv1 xenografts, shARV7 xenografts had a more robust response to enzalutamide, in that changes in tumor volume were greater in 22Rv1 xenografts with ARV7 knockdown [51]. These data suggest that ARV7 expression is negatively correlated with initial efficacy of enzalutamide in xenografts established with CR human cell lines [51]. The authors of this study also established enzalutamide-resistant LNCaP xenografts. First, LNCaP cells were transduced with an AR-FL expressing lentivirus and established in pre-castrated mice [51]. When tumors reached approximately 100 mm^3^, mice were treated with enzalutamide by oral gavage (10 mg/kg/day) [51]. LNCaP AR-FL overexpressing xenografts were very responsive to enzalutamide treatment, where treated tumors were decreased in volume by about 8-fold compared to control tumors after 28 days [51]. However, 2 LNCaP tumors eventually relapsed between 7 and 17 weeks of continuous enzalutamide treatment [51]. To generate enzalutamide-resistant LNCaP xenografts, these 2 relapsed tumors were resected and pieces of ~ 20 mm^3^ were transplanted subcutaneously into pre-castrated mice [51]. When resected LNCaP tumors reached 100-200 mm^3^, mice were treated with enzalutamide [51]. After continuous enzalutamide treatment, tumors that reached 800 mm^3^ were resected again and pieces transplanted [51]. Serial passaging of relapsing LNCaP tumors was continued in this way and tumors from passages 2-4 were considered to be enzalutamide-resistant [51]. Quantitative RT-PCR analysis of ARV expression showed that AR^v567es^ mRNA was significantly increased 11.9 times in enzalutamide-resistant LNCaP tumors compared to enzalutamide-sensitive tumors [51]. ARV7 expression was also increased in enzalutamide-resistant LNCaP tumors, but data only approached significance [51]. Unlike AR^v567es^ and ARV7, expression of ARV4 was unchanged in enzalutamide-resistant LNCaP tumors compared to enzalutamide-sensitive tumors [51]. Together these data show that expression of AR^v567es^, and possibly ARV7, is associated with acquired resistance to enzalutamide in AR-FL overexpressing LNCaP xenografts [51]. Again showing that despite the recent FDA approval of enzalutamide as a potent AR inhibitor for the treatment of CR-PCa, the persistence of ARV expression challenges the efficacy of this novel therapy.

A recent study from Dr. Dehm's laboratory has also demonstrated the role of AR^v567es^ in promoting resistance to enzalutamide [59]. As described in a previous section, R1-D567 is a CWR-R1-derived cell line engineered to exclusively express AR^v567es^ [58]. Considering that human PCa demonstrates both intratumoral and intertumoral molecular heterogeneity , Chan *et al*. performed a novel xenografting experiment with R1-D567 and parental R1-AD1 cells [59, 69]. Before grafting, a 90%/10% mixture of R1-AD1/ R1-D567 cells was prepared and this cell mixture was subcutaneously injected into intact mice [59]. After 28 days, 100 mm^3^ tumors were biopsied, mice castrated, and treatment began for 7 days with enzalutamide by oral gavage (30 mg/kg/day) [59]. After enzalutamide treatment, tumors were biopsied again and allowed to grow until another biopsy was performed after an additional 6 days [59]. Finally, tumors were harvested 2 days after the third biopsy [59]. Immunoblot analysis of protein lysates from the original 90%/10% R1-AD1/R1-D567 mixture, 3 sequential biopsies, and tissue at the experimental endpoint was performed with an antibody against AR NTD [59]. The lower molecular weight protein band representing AR^v567es^ was weak compared to the AR-FL band in lysates from the 90%/10% R1-AD1/R1-D567 mixture [59]. These results were expected, since cells exclusively expressing AR^v567es^ represented only 10% of lysed cells [59]. Similar ratios between AR-FL and AR^v567es^ protein bands were observed for lysates from the first biopsy obtained prior to castration and enzalutamide treatment [59]. However, after castration plus enzalutamide treatment, intensity of the AR^v567es^ band increased so that it was equivalent to AR-FL [59]. These data suggest that AR^v567es^ expression increases in response to castration and enzalutamide treatment, confirming the findings of other groups described above [51]. In addition, these data also suggest that AR^v567es^ may promote survival of tumor cells in an androgen-deprived environment [59]. AR^v567es^ increases may represent a shift where AR^v567es^ expressing R1-D567 cells make up a greater percentage of the tumor as a result of death of AR-FL expressing R1-AD1 cells [59]. AR^v567es^ protein expression maintained higher levels in lysates from the third biopsy (6 days after removal of enzalutamide) and in final tissue samples (8 days after removal of enzalutamide) [59]. At the same time, AR-FL protein expression decreased in these samples [59]. These data show that the original ratio of AR-FL to AR^v567es^ was not restored when enzalutamide treatment was terminated, indicating that even short-term treatment with second generation ADT agents can promote expression of ARVs that cannot be reversed [59].

### AR splice variants in patient-derived xenograft models of prostate cancer

The method described above utilized by Cao *et al*. to serially resect cell line-derived xenograft tumors is similar to the technique used to established PDX *in vivo* models [51]. Tumor samples isolated in the clinic are transplanted subcutaneously into an immunocompromised mouse—maintaining the original tissue architecture and cellular properties that arose during tumor progression in the patient [62]. The LuCaP series of clinical samples is the most highly utilized by researchers to establish PDX models of PCa, where LuCAP 23 and LuCaP 35 have been used by multiple research groups to study ARVs [70, 71]. In Dr. Dehm's original study that first described ARVs, LuCaP 23.1 and LuCaP 35, both isolated from lymph node metastases , were used to explore the role of AR^1/2/2b^ in tumor progression [43, 70, 71]. Specifically, both androgen sensitive and CR tissue bits, the later derived from relapsing xenografts exposed to long-term androgen deprivation , were used to establish LuCaP 23.1 and LuCaP 35 xenografts [43, 70, 71]. When mRNA expression of AR^1/2/2b^ was measured by quantitative RT-PCR, AR^1/2/2b^ transcripts were detectable in both LuCAP 23.1 and LuCaP 35 xenografts, showing for the first time that ARVs are present in patient-derived material [43]. In addition, AR^1/2/2b^ was increased in CR variant xenografts for both LuCAP 23.1 and LuCAP 35, indicating that ARVs could promote relapse in PCa patients treated with ADT [43]. Protein expression of ARVs, defined as bands between 60 and 80 kDa observed with two independent AR NTD antibodies, but not with an AR LBD antibody, was increased in CR xenograft tissue for both LuCaP 23.1 and LuCaP 35 [43]. In addition, while ARV protein expression could not be detected in androgen-sensitive LuCaP 23.1, it was present in androgen-sensitive LuCaP 35 tissue lysates [43]. These data suggest that the unique properties of the LuCaP 35 clinical samples may promote basal expression of ARVs without serial transplantation in the presence of ADT [43].

A more detailed study of castration recurrence in PDX models of PCa also demonstrated the link between ARV expression and resistance to ADT. Watson *et al*. established androgen sensitive LuCaP 35 xenografts in intact mice, allowed tumors to grow to 500-1000 mm^3^, castrated the mice, and harvested tissue 4 days after castration [53]. In addition, a separate group of mice with established LuCaP 35 xenografts that had been castrated for 3 days underwent testosterone replacement for 4 days [53]. Similar to the results from Dehm *et al*. that demonstrated basal expression of ARVs in LuCaP 35 xenografts , immunoblot with an AR NTD antibody of protein lysates from LuCaP 35 xenografts from intact mice showed bands between 60 and 80 kDa [43, 53]. Unlike previous studies, protein expression of ARVs was not increased in LuCaP 35 tumors from castrated mice compared to intact tumors [53]. However, ARV protein expression may not have been increased in this experiment because these mice were castrated for only 4 days [53]. Addition of testosterone decreased expression of ARVs in LuCaP 35 xenografts isolated from castrated mice—demonstrating that activation of ARV expression may potentially be reversible if testosterone is restored [53]. Indeed, testosterone treatment at supraphysiologic levels has been shown to decrease PSA levels in some advanced CR-PCa patients and it is possible that decreased expression of ARVs could be responsible for these responses [72].

Multiple other LuCaP models have also been shown to express ARVs. As described above, AR^v567es^ was first discovered by semi-quantitative PCR studies with primers against AR exon 2 and 8 (Figure 4A) using cDNA from LuCaP xenografts [47]. Sun *et al*. established that AR^v567es^ was expressed in 23 LuCAP xenografts, with tissue originating from 19 different patients [47]. These LuCaP xenografts represented a diverse array of source material, comprising tissue isolated from primary PCa and metastases from lymph node, liver, bone (femur and rib), bladder, bowel, peritoneum, and omental fat [47]. These data show that expression of AR^v567es^ is present in PCa tumors from multiple different organ microenvironments—demonstrating the prevalence of this variant [47]. Unfortunately, Sun *et al*. did not measure expression of ARV7 in this LuCaP panel, so it is unknown whether ARV7 expression is as widespread as AR^v567es^ in these PDX models. For those LuCaP models for which CR versions were also tested, including LuCaP 23.1, LuCaP 35, and LuCaP 96, AR^v567es^ expression was increased compared to androgen-sensitive LuCaP xenografts [47]. These data are consistent with Dehm *et al*. who showed that protein expression of ARVs was increased in CR xenografts of LuCAP 23.1 and LuCaP 35 [43]. Together, these data suggest that expression of ARVs is associated with development of resistance to ADT in patients and is thus considered one of the major pathways by which androgen- and AR-targeted therapies fail in advanced PCa [25].

### AR splice variants in patient-derived xenograft models of prostate cancer treated with abiraterone

As with human cell line-derived xenograft models of PCa, expression of ARVs are associated with second generation ADT treatment in PDX PCa models. Mostaghel et al. established xenografts using castration resistant lines of LuCaP 35 (LuCaP35CR) and LuCaP 23 (LuCaP23CR) in pre-castrated mice [73]. When tumors reached 250-300 mm3, mice were randomly assigned to daily vehicle or abiraterone (0.5 mmol/kg/day) treatment for 21 days [73]. For mice bearing either LuCaP23CR or LuCaP35CR tumors, abiraterone decreased serum PSA during the first 10 days of treatment, as expected [73]. In addition, abiraterone inhibited intratumoral production of androgens at early timepoints, starting at day 7 for both LuCaP23CR and LuCaP35CR tumors [73]. Abiraterone also decreased tumor growth, where median growth per day was significantly decreased in abiraterone-treated LuCaP23CR (5.0% per day vs. 7.4% per day) and LuCaP35CR (2.5% per day vs. 4.8% per day) [73]. Median survival of tumor-bearing mice was also improved by abiraterone, where survival for LuCaP23CR-bearing mice significantly increased from 14 to 24 days and survival for LuCaP35CR-bearing mice significantly increased from 17 to 39 days [73].

Despite the marked anti-tumor effect of abiraterone in these human PDX models of castration-resistant PCa, prolonged abiraterone treatment led to recurrence for some LuCaP23CR and LuCaP35CR tumors [73]. The authors classified these recurrent tumors into two groups, one including tumors that recurred and were resected within the 21-day abiraterone treatment regimen (Abi-R) and the other including tumors that recurred and were resected after the 21-day abiraterone treatment regimen (Abi-T) [73]. To understand the mechanism by which these tumors were acquiring resistance to abiraterone, mRNA expression of AR-FL, ARV7, AR^v567es^ was measured by quantitative RT-PCR on vehicle-treated, Abi-R, and Abi-T tumors from both LuCaP23CR and LuCaP35CR [73]. For LuCaP23CR and LuCaP35CR, AR-FL expression was significantly increased compared to vehicle in both Abi-R and Abi-T resistant tumors [73]. For ARV7, expression was significantly increased compared to vehicle in only Abi-T resistant tumors for LuCaP35CR, but was unchanged in both Abi-R and Abi-T for LuCaP23CR [73]. Conversely, expression of AR^v567es^ was significantly increased compared to vehicle in LuCaP23CR for both Abi-R and Abi-T, but increases in LuCaP35CR only approached signficance in Abi-R and Abi-T (p=0.078 and p=0.073, respectively) [73]. These data suggest that while increased expression of AR-FL was associated with resistance to abiraterone in both LuCaP23CR and LuCAP35CR tumors, expression of ARVs in abiraterone-resistant tumors was not as widespread. In addition, the results of this study indicate that ARV expression is associated with abiraterone resistance depends both on the specific PCa PDX model and timing of acquired abiraterone resistance.

### Genetically engineered mouse models and AR splice variants

Data from both cell line-derived xenograft and PDX models of PCa indicate that expression of ARVs occurs in vivo and is associated with castration recurrence. To understand the role of ARVs in development and progression of PCa, GEMMs have been developed that overexpress ARVs specifically in the prostate. Liu et al. created a prostate-specific AR^v567es^-overexpressing mouse by cloning an expression cassette with cDNA coding for human AR^v567es^ downstream of the prostate-specific probasin (Pb) promoter , microinjecting this cassette into fertilized C57BL/6 mouse embryos, and implanting embryos into pseudopregnant females [74, 75]. Once the transgenic Pb-AR^v567es^ GEMM was established, confirmation of AR^v567es^ overexpression was performed using quantitative RT-PCR with a Taqman probe targeted to the junction between AR exon 4 and 8 [75]. In addition, tissue expression of AR^v567es^ was shown in all four lobes of the mouse prostate using an AR NTD antibody that reacts only to human AR [75]. At 19 weeks of age, prostate weights were significantly increased in Pb-AR^v567es^ compared to wildtype mice [75]. In addition, Ki67 tissue staining, which marks dividing cells, was also increased in Pb-AR^v567es^ prostates [75]. These data suggest that forced expression of AR^v567es^ in the prostate increases cell proliferation [75]. Indeed, Pb-AR^v567es^ mice between 16 and 20 weeks of age develop prostatic hyperplasia, evidenced by an enlarged epithelial cell layer and increased cell number as shown by hemotoxlin and eosin (H&E) histological staining [75]. By 30-40 weeks of age, Pb-AR^v567es^ mice develop prostatic intraepithelial neoplasia (PIN), pre-malignant lesions that are characterized by elongated nuclei, prominent nucleoli, and overlapping cells [75]. Finally, Pb-AR^v567es^ mice at 1 year of age develop well-differentiated adenocarcinoma, defined histologically by increased number of glands, thickening of the stroma, inflammation, and hyperchromatic nuclei with increased mitosis [75]. These data show that development of adenocarcinoma is promoted by AR^v567es^ expression in prostatic epithelial cells [75]. To assess the effect of castration on prostatic adenocarcinoma in this model, Pb-AR^v567es^ mice at 50 weeks of age were castrated and prostate tissue analyzed following 3 weeks of castration [75]. Unlike intact Pb-AR^v567es^ mice, castrated Pb-AR^v567es^ mice develop invasive prostatic adenocarcinoma [75]. Histological changes included acini extending into the surrounding stroma and periprostatic connective tissue forming with evidence of inflammation [75]. These data show that overexpression of AR^v567es^ is oncogenic in the prostate and that castration promotes progression of adenocarcinomas that develop in this environment.

A similar GEMM that overexpresses ARV7 in prostatic epithelial cells has also been developed by Sun et al. [76]. Human cDNA for ARV7 was cloned downstream of the Pb promoter, expression cassette microinjected into fertilized FVB mouse embryos, and embryos implanted into pseudopregnant females [76]. Expression of ARV7 in transgenic mice was confirmed using semi-quantitative PCR of genomic DNA with ARV7-specific primers [76]. While confirmation of ARV7 expression in prostates was carried out by immunoblot using an ARV7-specific antibody [76]. These data show that the expression cassette was incorporated into the genome and that ARV7 protein overexpression in the prostate was established [76]. In addition, tissue expression of ARV7 was confirmed using immunohistofluorence on frozen prostate sections with an ARV7-specific antibody [76]. By 1 year of age, H&E staining showed that Pb-ARV7 mice develop PIN lesions in the dorsolateral prostate, which also show strong expression of ARV7 by immunohistochemistry [76]. In addition, PIN lesions were also positive for Ki67, indicating that cell proliferation is increased in Pb-ARV7 mice [76]. These data suggest that ARV7 overexpression in prostate epithelial cells promotes development of pre-malignant lesions [76]. Since the authors do not offer histological data after 1 year, it is unknown whether Pb-ARV7 mice progress to prostatic adenocarcinoma as they further increase in age. Together, histological data from Pb-AR^v567es^ and Pb-ARV7 GEMMs suggest that ARVs can promote development of PIN lesions and in the case of AR^v567es^, progression to prostatic adenocarcinoma [75, 76]. Pb-AR^v567es^ mice develop PIN at 30-40 weeks of age and Pb-ARV7 mice develop PIN at 1 year of age, suggesting that AR^v567es^ is more oncogenic than ARV7; however, these data may only be the result of mouse strain differences, since Pb-AR^v567es^ mice were established on the C57BL/6 background and Pb-ARV7 mice were established on the FVB background [75, 76]. In addition, it is unclear how these models translate to human disease since neither Pb-AR^v567es^ nor Pb-ARV7 GEMMs were reported to develop metastases -not altogether surprising since metastasis rarely occurs in PCa GEMMs [77]. The majority of human PCa cell lines and the LuCaP tissue series, most of which are derived from metastases, only express detectable levels of ARVs after prolonged castration. Whereas, AR^v567es^ and ARV7 transgenes in these GEMMs are activated between 2 and 7 weeks of age when under the control of the Pb promoter and overexpression is maintained throughout the lifetime of the mouse [74]. While this inherent difference in expression pattern raises questions about the ability to compare lesions that develop in Pb-AR^v567es^ and Pb-ARV7 GEMMs to human PCa, these studies offer important data to suggest that ARVs can promote tumor development in the prostate.

## CLINICAL UTILITIES OF AR SPLICE VARIANTS

### AR splice variant expression in clinical samples and in response to therapy

Pre-clinical models have been imperative to our understanding of molecular mechanisms that drive PCa. However, human xenografts may not maintain pathological characteristics of the tumors from which they are derived and while there are GEMMs which develop PCa, mice are particularly resistant to developing advanced disease and mouse tumors often do not simulate human PCa [64, 77]. Clinical investigation of molecular findings from human xenografts and GEMMs is essential for a more complete understanding of mechanisms that drive PCa and promote ADT resistance. Accordingly, multiple studies have focused on defining ARV expression patterns in human PCa tumor tissue samples at various stages of disease. In the first study to show ARV expression in clinical samples, Hu et al. performed semi-quantitative RT-PCR for ARV1 and ARV7 and found that both were increased in CR-PCa tumors compared to hormone-naïve PCa tumors [44]. When expression of ARV7 was assessed by quantitative RT-PCR in 124 clinical samples, both hormone-naïve and CR-PCa samples had increased expression of ARV7 compared to normal prostate tissue [44]. In addition, expression of ARV7 in CR-PCa samples was increased compared to hormone-naïve PCa samples, confirming results from semi-quantitative RT-PCR [44]. When patients who had undergone prostatectomy were grouped according to ARV7 expression levels, data showed that those with ARV7 less than median had significantly increased progression-free survival, defined by serum PSA levels, compared to patients with ARV7 greater than median [44]. These data were the first to suggest that ARV7 expression could be used as a biomarker to identify those patients who are most likely to fail therapy. In contrast to ARV7, ARV1 expression levels in tumor tissue from prostatectomy patients were not predictive of PSA recurrence [44]. Guo et al. measured tissue expression of ARV7 by immunohistochemistry with an ARV7-specific antibody in benign prostate and in PCa tumors from both hormone-naïve and CR-PCa patients [45]. ARV7 showed minimal staining of the basal and stromal cells in benign tissue, but luminal cells were negative [45]. In hormone-naïve tissue, luminal cells showed strong cytoplasmic staining of ARV7, while in CR-PCa tissue, ARV7 staining in luminal cells partially shifted to the nucleus [45]. These data suggest that cell type patterns of ARV7 expression change with the development of prostatic adenocarcinoma [45]. In addition, intracellular location of ARV7 is distinctive in CR-PCa compared to hormone-naïve tumors [45]. Since immunohistochemistry shows that ARV7 appears to move to the nucleus in CR-PCa tissue, these data suggest that ARV7 activity is activated when patients become resistant to ADT, confirming the findings of many studies in CR cell culture models. When cytoplasmic and nuclear staining of ARV7 was assessed quantitatively, hormone-naïve and CR-PCa tumors had significantly increased ARV7 staining in both the cytoplasm and the nucleus compared to benign tissue [45]. In turn, cytoplasmic and nuclear staining for ARV7 was significantly increased in CR-PCa compared to hormone-naïve tumors [45]. Consequently, the authors hypothesized that ARV7 staining could be associated with clinical outcome. Indeed, cytoplasmic staining intensity of ARV7 correlated with PSA recurrence after prostatectomy, providing further evidence to support the use of ARV7 as a biomarker [45].

In the two studies described above, benign and PCa tumor specimens were isolated from different patients, making it possible that the differences observed in ARV expression are the result of individual differences between patients and not PCa progression. To address this question, Sun et al. isolated matched tissue samples at prostatectomy for a limited amount of patients from benign and malignant regions of the prostate using laser capture [47]. Semi-quantitative RT-PCR for ARV7 and AR^v567es^ in these matched benign and primary PCa tumor specimens showed that ARV expression was present in the adenocarcinoma and not the benign regions in 2 out of 8 (25%) patients [47]. These data suggest that ARV expression is increased as a patient progresses to malignant prostate adenocarcinoma; however, since the patients from which matched samples were obtained had never undergone ADT, these data do not address the role of castration in ARV expression in individual lesions. The authors aimed to understand the contribution of ADT and therapy resistance to ARV expression by acquiring CR-PCa tumor samples at autopsy [47]. Amplification of AR-FL, ARV7, and AR^v567es^ transcripts by quantitative RT-PCR with cDNA made using 69 metastatic CR-PCa tumor samples from 13 patients showed that 46 samples were positive for AR-FL or either ARV [47]. Surprisingly, expression of AR was not detectable in 23 samples, but since these 23 CR-PCa samples were isolated from patients with primary tumors with neuroendocrine phenotype, an AR-negative PCa variant, undetectable AR expression was anticipated [47, 78]. Of the 46 CR-PCa metastases which were AR-positive, 27 out of 46 (58.6%) expressed at least one ARV, 15 (32.6%) expressed just ARV7, 20 (43.4%) expressed just AR^v567es^, and 6 (13%) expressed both ARV7 and AR^v567es^ [47]. Whereas, quantitative RT-PCR using cDNA isolated from 35 normal prostate tissue samples showed that 10 out of 35 (28.5%) expressed at least one ARV, 4 (11.4%) expressed ARV7, 6 (17.1%) expressed AR^v567es^, and none expressed both ARV7 and AR^v567es^ [47]. These data suggest that expression of ARVs are associated with resistance to ADT in PCa patients, providing clinical support for the positive relationship found between ARV expression and castration in human xenograft models.

ARV expression data described above from CR-PCa patient autopsies are endpoint measurements; therefore it is unclear from these results whether ARV expression can predict disease progression in CR-PCa patients. To assess the prognostic value of ARV expression, Hornberg et al. collected bone metastatic samples from 30 CR-PCa patients during orthopedic surgery for metastatic spinal cord compression [54]. Quantitative RT-PCR for ARV7 and AR^v567es^ was performed in these bone metastatic samples to stratify patients based on ARV expression [54]. The 7 CR-PCa patients with ARV7 expression in the upper quartile had significantly decreased cancer-specific survival compared to the 23 patients in the lower 3 quartiles [54]. For those CR-PCa patients who had detectable expression of AR^v567es^ (n=7), cancer-specific survival was not significantly decreased compared to patients lacking AR^v567es^ (n=23); however, diminished survival for AR^v567es^-expressing patients trended toward significance [54]. Finally, when these two metrics were combined, the 10 CR-PCa patients with ARV7 expression in the upper quartile and/or detectable expression of AR^v567es^ had decreased cancer-specific survival compared to the 20 patients who did not [54]. These data suggest that ARV expression is associated with more life-threatening disease in CR-PCa patients [54]. This study shows for the first time that ARV expression predicts development of lethal CR-PCa, indicating that ARVs can serve as biomarkers for identifying those PCa patients most at risk for death [54].

To study the role of ARV7 expression in response to enzalutamide treatment of CR-PCa patients, Efstathiou et al. performed a novel prospective Phase II trial (NCT01091103, https://clinicaltrials.gov/) [79]. Patients with bone metastatic CR-PCa underwent transilial bone marrow biopsies prior to and after 8 weeks of enzalutamide treatment and ARV7 tissue expression in bone marrow infiltrating tumor cells was assessed by immunohistochemistry [79]. The effectiveness of enzalutamide was measured by monitoring serum PSA and imaging, separating patients into response groups [79]. Those patients who displayed primary resistance to enzalutamide did not show PSA decline or imaging improvement within 4 months of treatment [79]. For those patients who benefited from enzalutamide, moderate responders showed PSA decline and imaging improvement within the first 4 months of treatment, but progressed between 4 and 6 months of treatment [79]. Finally, prolonged responders showed no evidence of disease progression within at least 6 months of treatment [79]. Expression of ARV7 in tumor cells from bone marrow of patients before treatment did not predict responsiveness to enzalutamide [79]. In that, the number of patients with detectable ARV7 in the resistance group was not significantly different compared to the number of patients with detectable ARV7 in both benefit groups (moderate and prolonged) [79]. However, for the samples collected after 8 weeks enzalutamide treatment, the number of patients with detectable expression of ARV7 in the resistance group was significantly increased compared to the number of patients with detectable expression of ARV7 in the benefit groups [79]. In addition, no patients who experienced prolonged benefit from enzalutamide treatment showed expression of ARV7 in tumor cells from bone marrow aspirate either at baseline or after 8 weeks [79]. This study was the first to collect bone metastatic samples from CR-PCa patients who are undergoing treatment with a second generation ADT agent [79]. In addition, these data show for the first time that ARV7 expression in bone marrow metastases can be predictive of CR-PCa patient responsiveness to enzalutamide [79]. However, it is important to note that while ARV7 expression may not be able to determine which CR-PCa patients should undergo enzalutamide treatment, it is perhaps an effective biomarker for those patients who should continue treatment [79].

Less well-studied ARVs have also been detected in PCa tumor specimens. One study showed AR23 to be expressed in CR-PCa, where 5 of 8 metastatic CR-PCa tumors from patients treated with AR antagonists contained the 69 nucleotide sequence unique to AR23 (Figure 3), which was not observed in any of the metastatic tumors from 3 hormone-naïve patients [80]. However, this study did not assess whether AR23 expression correlated with clinical characteristics of these CR-PCa cases [80]. When ARV9 and ARV12 were analyzed in normal prostate, hormone-naïve PCa, and CR-PCa tumor samples, quantitative RT-PCR showed that expression of both ARV9 and ARV12 were significantly increased in CR-PCa tumor specimens compared to both normal prostate and hormone-naïve PCa [49]. Neither ARV9 nor ARV12 expression correlated with pre-operative PSA, Gleason score, or tumor stage ; therefore, it is unclear whether these specific variants contribute to disease progression or could serve as clinical biomarkers [49].

Recently, whole transcriptome analysis of metastatic CR-PCa biopsies from living individuals determined ARV expression to be widely distributed [81]. Using NGS, read numbers for AR exon junctions were assessed in 125 CR-PCa patients [81]. Unsurprisingly, the percentage of exon 3-CE3 junction reads (ARV7) to exon 1-2 junction reads (AR-FL and all ARVs) was highest compared to read percentages for other ARVs [81]. These data provide further proof that ARV7 is the most abundant ARV expressed in human cell lines, human xenografts, and clinical samples. The authors question the significance of ARVs in CR-PCa, since they show that variants are also expressed in benign prostate tissue and primary PCa by mining NGS data in The Cancer Genome Atlas (TCGA) database [81]. However, traditional molecular methods performed in multiple studies from independent laboratories described in this review show that ARV expression correlates with development of ADT resistance in numerous model systems and clinical samples. Overall, the clinical data discussed in this section suggest that ARV expression could be an effective biomarker for lethal CR-PCa and progression at multiple stages of disease.

### AR splice variants as biomarkers for advanced prostate cancer and therapeutic efficacy

Recent work on ARVs in PCa has focused on exploiting the correlative value of ARV expression with disease aggressiveness and progression as clinical biomarkers. In 2014, Antonarakis et al. published an influential prospective study in the New England Journal of Medicine that measured mRNA expression of ARV7 in circulating tumor cells (CTCs) from metastatic CR-PCa patients before starting treatment with enzalutamide or abiraterone [82] (Table 4). CTCs are tumor cells that have entered the bloodstream of patients with solid tumors [83]. It is estimated that a 7.5 mL sample of blood has 1 CTC per 10^6^ white blood cells (WBCs), emphasizing the rarity of these cells and the requirement for cell selection [83]. In this study, 30 mL peripheral whole blood was collected by venipuncture and samples submitted to positive selection for PCa CTCs using the AdnaTest ProstateCancerSelect kit [82] (Table 4). CTCs were isolated using immunomagnetic beads coated with three antibodies against proteins located on the extracellular surface of the PCa cell membrane, with one antibody against epithelial cell adhesion molecule (EpCAM) and the other two antibodies being proprietary [82]. In the second phase of the kit referred to as ProstateCancerDetect, isolated cells were lysed and RNA isolated [82]. Magnetic beads coated with oligo-dT were used to purify mRNA and cDNA was produced by reverse transcription [82]. Quantitative RT-PCR was performed for AR-FL and ARV7 with cDNA generated using ProstateCancerDetect from patients before their treatment regimens with either enzalutamide or abiraterone, where AR-FL RT-PCR was used as a positive control [82]. Of the 31 patients who received enzalutamide, 39% had CTCs that were positive for ARV7 and these patients had significantly lower PSA response rates (0% vs. 53%) [82]. ARV7-postive patients treated with enzalutamide had shorter median overall survival (5.5 months), whereas overall survival could not be calculated for the ARV7-negative group since several patients were still alive at the end of the study [82]. ARV7 expression was detected in CTCs for 19% of 31 abiraterone-treated patients, where ARV7-positive patients also had significantly lower PSA response rates (0% vs. 68%) [82]. Median overall survival was lower for ARV7 positive patients treated with abiraterone (10.6 months) compared to ARV7 negative patients, for which overall survival again could not be determined since several patients were still alive at the end of the study [82]. Together, these data show that ARV7 expression in CTCs can predict a patient's response to either enzalutamide or abiraterone [82]. For a small number of patients in this study, PCa metastatic tissue specimens were collected either at autopsy or by core needle biopsies [82]. Using these samples, RNA in situ hybridization for ARV7 was performed to confirm that detection of ARV7 in CTCs reflects expression in solid tumors [82]. Indeed, for the 3 patients for which data was presented, in situ hybridization showed that ARV7 mRNA was present in metastatic tumors from 2 patients whose CTCs were positive for ARV7, whereas ARV7 mRNA was not detected in a metastatic tumor specimen from 1 patient whose CTCs were negative for ARV7 [82]. These data suggest that expression levels of ARV7 detected in CTCs match those in metastatic tumors, emphasizing the validity of measuring ARV7 expression in CTCs by “liquid biopsy” [82].

**Table 4:** Biomarker Development for Androgen Receptor Splice Variants in Liquid Biopsy

Method	Study Type	Transcript (s)	Associated with Disease / Therapy	Blood Volume (mL)	References
CTC Positive Selection	Prospective	ARV7	Yes	30	[82]
CTC Positive Selection	Prospective	ARV7	Yes	7.5	[84]
CTC Negative Selection	Cross-sectional	ARV7, AR^v567es^	Not measured	10	[85]
Whole Blood RNA	Cross-sectional	ARV7, AR^v567es^	Yes	5	[85]
Whole Blood RNA	Prospective	ARV7	Yes	2.5	[86]
Whole Blood RNA	Retrospective	ARV7, PSA	Yes	7	[87]
CTC Positive Selection	Retrospective	ARV7	No	7.5	[89]

CTC: Circulating tumor cells; AR: Androgen receptor; PSA: Prostate specific antigen

In another prospective study, Steinestel et al. used AdnaTest ProstateCancerSelect to isolate PCa CTCs from 7.5 mL of blood collected from patients at multiple stages of disease preparing to switch to new therapy following PSA progression [84] (Table 4). ARV7 expression was measured by quantitative RT-PCR in CTCs, showing that 18 out of 37 patients (48.6%) had detectable ARV7 [84]. No patients who were hormone-naïve harbored CTCs that were positive for ARV7 , as expected since both preclinical and clinical studies have shown expression of ARVs is correlated with androgen deprivation [84]. After patients switched to new therapies, which included primary ADT, enzalutamide, abiraterone, or docetaxel, 10 out of 14 (71.4%) patients with ARV7-negative CTCs showed > 50% reduction in PSA levels during treatment, while only 1 out of 15 (6.6%) patients with ARV7-positive CTCs experienced this biochemical response to therapy [84]. These results suggest ARV7 negativity in CTCs identifies patients who will respond when a new therapeutic regimen is implemented , supporting the results from Antonarakis et al. [82, 84]. It is important to note that the AdnaTest ProstateCancerSelect kit is estimated to capture 1 PCa CTC within 5 mL of blood along with non-specific isolation of 1000 WBCs [82]. Since Antonarakis et al. collected 30 mL of blood from each patient and Steinestel et al. collected 7.5 mL (Table 4), cDNA was produced using approximately 6 PCa CTCs and 6000 WBCs or 1 PCa CTC and 1500 WBCs, respectively [82]. As reported in the supplementary material of Antonarakis et al., when only 5 VCaP cells were spiked into 5 mL of whole blood from healthy donors and the AdnaTest ProstateCancerSelect protocol was carried out, semi-quantitative RT-PCR for ARV7 detected an amplicon not present in whole blood without VCaP cells [82]. Therefore, ARV7 mRNA expression is capable of being detected in 5 CTCs isolated by ProstateCancerSelect [82]. However, these details emphasize the analogy stressed in a recent review article published by Dr. Kenneth Pienta's group that collecting CTCs is “like finding a needle in a haystack” [83]. Therefore, the practicality of using CTCs in PCa therapeutic decision-making remains questionable.

Considering the extremely low likelihood of isolating PCa CTCs, Liu et al. recently carried out a study to compare results for detection of ARV7 and AR^v567es^ by RT-PCR using RNA isolated from either PCa CTCs or whole blood [85]. The authors emphasize that positive selection of CTCs using specific antibodies against epithelial markers, like that used by Antonarakis et al. and Steinestel et al. (Table 4), might exclude CTCs that have undergone epithelial-to-mesenchymal transition [82, 84, 85]. Therefore, PCa CTCs are isolated in this study using negative selection, where WBCs were removed from 10 mL of whole blood using immunomagnetic beads with an antibody against CD45 [85] (Table 4). Thus, CD45-negative cell populations were considered to be PCa CTCs [85]. In parallel, RNA was directly isolated from 5 mL of whole blood using the PAXgene Blood RNA kit [85] (Table 4). To compare these two methods, CTC negative enrichment and direct RNA isolation was carried out using samples from 10 CR-PCa patients and quantitative RT-PCR was performed for both ARV7 and AR^v567es^ [85]. While both methods detected ARV7 in 9 out of 10 patients, ARV7 levels were approximately 40% lower using RNA from CTC negative selection compared to direct RNA from whole blood when samples from the same patient were processed side-by-side [85]. These data suggest that isolation of CTCs promotes loss of sensitivity in detection of ARV7 expression by RT-PCR from liquid biopsy [85]. In addition, while AR^v567es^ expression was detected in 2 out of 10 patients using RNA from whole blood, expression of AR^v567es^ was detected in only 1 out of 10 patients using RNA from CTC negative selection [85]. These data suggest that CTC enrichment is also inferior to RNA isolation from whole blood in measurement of AR^v567es^ expression [85]. Based on these results, the authors chose to use the whole blood RNA approach in a cross-sectional study of 73 samples from 46 CR-PCa patients to assess validity [85]. Quantitative RT-PCR showed that AR-FL was expressed in 69 out of 73 samples (94.5%), ARV7 was expressed in 50 samples (68.4%), and AR^v567es^ was expressed in 23 samples (31.5%) [85]. In addition, 53 out of 73 samples expressed at least one ARV (72.6%) and 20 (27.3%) expressed both ARV7 and AR^v567es^ [85]. As expected based on known positive association between ARV7 expression and ADT treatment, ARV expression in whole blood was associated with history of treatment with second generation ADT [85]. Where 17 out of 25 samples (68.0%) from CR-PCa patients who received second-line ADT were positive for ARV7 and 9 out of 25 (36.0%) were positive for AR^v567es^ [85]. In comparison, ARV7 was expressed in only 3 out of 13 samples (23.1%) and AR^v567es^ was expressed in 0 out of 13 samples from CR-PCa patients who had not received second-line ADT [85]. Since this study did not prospectively assess ARV expression and response of CR-PCa patients to second generation ADT, it is unclear from these data if this whole blood RNA method of detecting ARV expression predicts treatment efficacy in these patients [85]. Nevertheless, this study shows for the first time in a direct comparison that RNA isolation from whole blood is more sensitive in detecting ARV expression by liquid biopsy than PCa CTC enrichment [85].

Two more recent studies have also assessed ARV expression analysis using RNA from whole blood of advanced PCa patients. In Todenhöfer et al., 2.5 mL blood was collected from 37 CR-PCa patients prior to treatment with abiraterone and quantitative RT-PCR was performed for ARV7 using RNA isolated from whole blood [86] (Table 4). None of the patients positive for ARV7 had a PSA response rate of ≥ 30% decrease after abiraterone treatment and 52% of patients negative for ARV7 showed ≥ 30% decrease in PSA; but these strong trends did not reach significance [86]. However, patients who were ARV7-positive had significantly decreased PSA progression-free survival with abiraterone compared to ARV7-negative patients (1.2 vs. 4.0 months) [86]. These data show for the first time that measuring ARV7 expression using RNA from whole blood has predictive value for CR-PCa patients being treated with abiraterone [86]. In addition, this study emphasizes that CTC enrichment may not be necessary for ARV7 to act as a biomarker [86]. In a retrospective study, Qu et al. identified CR-PCa patients who had been treated with either abiraterone (n=81) or enzalutamide (n=51) for which RNA samples from blood mononuclear cells in 7 mL samples were available [87] (Table 4). Quantitative analysis of ARV7 and PSA mRNA expression was measured using droplet digital PCR , a method where nucleic acids are partitioned into droplets in a water-oil emulsion and individual PCR reactions are performed in each droplet [87, 88]. Thereby, droplet digital PCR quantifies nucleic acids from a small amount of starting material in a precise manner [88]. When patients were ranked according to ARV7 expression, the upper third with ≥ 19 copies/μg RNA had shorter time to treatment failure compared to the lower two thirds for both abiraterone (8.0 vs. 15.6 months) and enzalutamide (3.6 vs. 5.6 months) [87]. Similarly, overall survival following treatment with abiraterone or enzalutamide was also significantly decreased in patients with high ARV7 expression compared to ARV7-low patients; where median survival was 27.2 vs. 35.6 months for abiraterone and 13.8 vs. 29.1 months for enzalutamide [87]. When PSA expression (positive or negative) was analyzed with ARV7 expression levels, its incorporation was additive in predicting those patients at risk for treatment failure or death following abiraterone or enzalutamide therapy [87]. CR-PCa patients treated with abiraterone who were ARV7-high and PSA-positive had a median of 5.6 months to treatment failure, which was significantly decreased compared to 8.1 months for ARV7-low and PSA-positive patients and 21.1 months for PSA-negative patients [87]. Median time to treatment failure for enzalutamide-treated CR-PCa patients was also significantly shorter for ARV7-high and PSA-positive patients compared to ARV7-low and PSA-positive and PSA-negative patients (2.8 vs. 3.0 and 13.4 months, respectively) [87]. The significant additive effect of PSA expression to ARV7 stratification of CR-PCa patients was also observed in analysis of median overall survival following treatment with abiraterone or enzalutamide [87]. ARV7-high and PSA-positive CR-PCa patients treated with abiraterone had a median overall survival of 21.3 months, while ARV7-low and PSA-positive patients had overall survival of 34.4 months and PSA-negative patients had overall survival of 43.2 months [87]. Enzalutamide-treated CR-PCa patients who were PSA-negative or ARV7-low and PSA-positive had median overall survival of 29.4 and 20.4 months, respectively, whereas ARV7-high and PSA-positive patients had significantly decreased overall survival at 12.5 months [87]. These data show that measuring expression of both ARV7 and PSA in whole blood RNA could provide more predictive value for treatment efficacy with second generation ADT in CR-PCa patients than ARV7 expression alone; however, prospective studies are required to assess the validity of these data [87].

The studies described above show that ARV7 is a potential biomarker for CR-PCa patient response to additional treatment regimens. However, a recent study published by Bernemann et al. presents results that conflict with the supposition that ARV7-expressing patients have inferior responses to second generation ADT [89]. In this group's previous study by Steinestel et al. described above (Table 4), one CR-PCa patient who had CTCs positive for ARV7 had a > 50% decrease in serum PSA after treatment with abiraterone [84]. These data led the authors to conduct a retrospective study where 21 patients with ARV7-positive CTCs prior to therapeutic regimens were specifically assessed for treatment response to either enzalutamide or abiraterone [89]. The authors considered that patients benefited from therapy if their disease remained stable, defined as < 50% decrease to < 25% increase in serum PSA [89]. While response to therapy was defined as > 50% decrease in PSA [89]. With these parameters, analysis showed that 6 out of 21 patients (28.6%) with ARV-positive CTCs derived benefit from therapy with either enzalutamide or abiraterone [89]. Surprisingly, 4 out of 6 ARV7-positive patients who benefited from second generation ADT responded with > 50% decrease in PSA [89]. These data are in direct opposition to previous studies showing that patients with ARV7-positive liquid biopsies, either via CTC enrichment or direct RNA isolation, are non-responders to second generation ADT [82, 84, 86]. When additional tumor and clinical data were analyzed, there were no significant differences found between ARV7-positive benefiting patients and non-benefiting patients for age, Gleason score, and length of time for which patients were stable on first generation ADT [89]. Although low sample size deters widespread conclusions based on this study, presentation of these data has provided a realistic view of the practicality of using ARV7 expression by liquid biopsy to make treatment decisions. Indeed, the authors emphasize that their results show that ARV7-positive status should not exclude CR-PCa patients from treatment with second generation ADT [89]. Less stringent measures of therapeutic benefit, such as defining stable disease as a benefit of therapy, are used in this study than in previously published research [89]. However, the authors draw attention to a single ARV7-expressing patient from Antonarakis et al. who experienced a ~ 30% decrease in serum PSA when treated with enzalutamide [82]. It is possible that assessing expression of ARV7 in CTCs, shown to be a less sensitive method of measuring ARV7 expression by liquid biopsy , is a factor that led to overlooking the ARV7-positive responsive group in previous studies [85]. For instance, direct isolation of RNA from whole blood and subsequent droplet digital PCR, as performed in Qu et al. , may have derived a range of ARV7 expression levels and found that the ARV7-positive patients who benefited from second generation ADT were those with the lowest levels of ARV7 [87]. In addition, it may be necessary to measure multiple biomarkers per liquid biopsy, as done by Qu et al. in measuring both ARV7 and PSA (Table 4), to determine those ARV7-positive CR-PCa patients who will respond to additional rounds of second generation ADT [87]. Speculation aside, the scientific authenticity of this small retrospective study is notable, especially since their findings conflict with their own published data , as well as the overwhelming majority of publications on ARV7 biomarker development from other laboratory groups [82, 84–87] (Table 4). Overall, measurement of ARV7 expression in liquid biopsy shows promise as a clinical biomarker for CR-PCa patient response to second-generation ADT, but several questions remain as to the true utility of this analysis. Indeed, as of this writing, there are 6 clinical trials either directly measuring the effectiveness of ARV7 in liquid biopsy as a predictive biomarker or using ARV7 expression as a parameter to assess response to novel therapies for CR-PCa (NCT02601014, NCT02438007, NCT02269982, NCT02853097, NCT02491411, NCT02429193, https://clinicaltrials.gov/). It is the hope that with the additional information generated by these trials that a definitive conclusion can be made as to whether the use of ARV7 as a biomarker is a valuable enough tool to universally implement for clinical decision-making.

## CONCLUDING REMARKS

The progression of evidence surrounding the role of ARVs in development of resistance to ADT in PCa represents a true example of translational uro-oncology research. Over nearly a decade, data defining ARV splice patterns, expression, transcriptional activity, DNA-binding potential, negative relationship to androgen levels, and promotion of tumor growth in pre-clinical models have built a schema that has founded the current efforts in ARV biomarker development. While accurate identification of non-responsive CR-PCa patients prior to treatment with ARV7-based assays exemplifies substantial progress, we lack targeted treatment options for these patients. Despite the depth of work that has been done studying the molecular biology of ARVs, small molecules have yet to be FDA-approved that specifically inhibit ARVs [13]. The next phase in ARV-focused research entails the discovery or development of ARV-targeted therapies. One of the strategies that has been employed has been targeting the NTD, since nearly all ARVs retain their NTDs following alternative splicing (Figure 3) and the NTD is required for transcriptional activity [90]. The EPI class of compounds directly bind to activating function domain 1 (AF1) located within the NTD of AR [90]. EPI-001 hinders androgen dependent growth in both androgen-sensitive, ARFL-expressing LNCaP xenografts but had no effect on ARFL-negative PC3 xenografts, indicating the specificity of EPI-001 to AR-expressing tumors [91]. In addition, EPI-001 specifically inhibits the transcriptional activity of AR^v567es^ in vitro [92]. EPI-002, a potent stereoisomer of EPI-001, inhibits growth of CR-PCa, ARV-expressing VCaP xenografts under castration conditions, indicating the ability of EPI compounds to inhibit ARVs in vivo [92]. The clinically active drug in this class, EPI-506, was derived from EPI-002 and is currently in Phase I/II clinical trial (NCT02606123, https://clinicaltrials.gov/) [90]. In this study, the Phase II component will include CR-PCa patients that are either post-abiraterone/enzalutamide-naïve, post-enzalutamide/abiraterone-naïve, or post-abiraterone/post-enzalutamide. However, patients will not be selected based on ARV7 status; instead, responses to treatment will be stratified based on ARV7 expression in CTCs as another outcome measure for the trial. At the time of this writing, the EPI-506 trial is currently recruiting and is slated to be completed by December 2017.

Another strategy that aims to target ARVs includes inducing their protein degradation. Indeed, recent work by Dr. Allen C. Gao's laboratory has suggested that promoting protein degradation of ARVs may provide clinical benefit in overcoming primary resistance to second generation ADT [93, 94]. In these studies, treatment with niclosamide, an antihelminthic drug FDA-approved in 1996, decreases protein levels of ARV7 in CR-PCa cell lines by promoting its protein degradation by the ubiquitin proteasome system [93]. In addition, cell line-derived xenograft models showed that addition of niclosamide to either enzalutamide or abiraterone significantly decreases tumor volume compared to either ADT agent alone, suggesting that niclosamide treatment can restore responsiveness to second generation ADT [93, 94]. Consequently, there are currently two clinical trials assessing the effectiveness of niclosamide in CR-PCa patients in combination with either enzalutamide (Phase I, NCT02532114) or abiraterone (Phase II, NCT02807805, https://clinicaltrials.gov/). In addition, a novel drug called galaterone, which both inhibits androgen synthesis and promotes degradation of AR-FL and ARV7 [50, 95–97], is in Phase III trials for treatment of ARV7-positive CR-PCa patients (NCT02438007, https://clinicaltrials.gov/). Together, outcomes from these clinical trials may show that targeting the AR NTD and/or enhancing protein degradation of ARV7 are viable strategies for ARV7-positive CR-PCa patients. Such a conclusion would provide absolute justification for the research enterprise in basic molecular biology, since the purely scientific benchwork that identified ARVs will have culminated in creating a curative treatment for CR-PCa patients otherwise non-responsive to therapy.

## Abbreviations

ADT: Androgen deprivation therapy
AF1: Activating function domain 1
AR: Androgen receptor
ARE: Androgen response element
AR-FL: Androgen receptor full length
ARG: Androgen response gene
ARV: Androgen receptor variant
BiFC: Bimolecular fluorescence complementation
BLAST^®^: Basic local alignment search tool
CE: Cryptic exon
ChIP: Chromatin immunoprecipitation
ChIP-seq: Chromatin immunoprecipitation-sequencing
CR-PCa: Castration-resistant prostate cancer
CT: Computated tomography
CTCs: Circulating tumor cells
DBD: Androgen receptor DNA binding domain
DHT: Dihydrotestosterone
EGF: Epidermal growth factor
EpCAM: Epithelial cell adhesion molecule
GEMM: Genetically-engineered mouse model
HD: Androgen receptor hinge domain
H&E: Hemotoxlin and eosin
LBD: Androgen receptor ligand binding domain
MMTV: Mouse mammary tumor virus
MRI: Magnetic resonance imaging
NGS: Next-generation sequencing
NTD: Androgen receptor N-terminal domain
Pb: Probasin
PCa: Prostate cancer
PDX: Patient-derived xenografts
PIN: Prostatic intraepithelial neoplasia
RACE: Rapid amplification of cDNA ends
RT-PCR: Real-time PCR
SLASR: Selective linear amplification of sense RNA
TCGA: The Cancer Genome Atlas
WBC: White blood cell
